# Finasteride Alleviates High Fat Associated Protein-Overload Nephropathy by Inhibiting Trimethylamine N-Oxide Synthesis and Regulating Gut Microbiota

**DOI:** 10.3389/fphys.2022.900961

**Published:** 2022-08-15

**Authors:** Zuoyuan Wang, Li You, Yuan Ren, Xiaoye Zhu, Xiaoyi Mao, Xiaowan Liang, Tingting Wang, Yumeng Guo, Te Liu, Jun Xue

**Affiliations:** ^1^ Division of Nephrology of Huashan Hospital, Fudan University, Shanghai, China; ^2^ Institute of Digestive Disease, Huashan Hospital, Fudan University, Shanghai, China; ^3^ Shanghai Geriatric Institute of Chinese Medicine, Shanghai University of Traditional Chinese Medicine, Shanghai, China

**Keywords:** trimethylamine N-oxide, gut microbiota, chronic kidney disease, Inflammtory, Finasteride

## Abstract

Unhealthy diet especially high-fat diet (HFD) is the major cause of hyperlipidemia leading to deterioration of chronic kidney diseases (CKD) in patients. Trimethylamine N-oxide (TMAO) is a gut-derived uremic toxin. Our previous clinical study demonstrated that the elevation of TMAO was positively correlated with CKD progression. Finasteride, a competitive and specific inhibitor of type II 5a-reductase, has been reported recently to be able to downregulate plasma TMAO level thus preventing the onset of atherosclerosis by our research group. In this study, we established a protein-overload nephropathy CKD mouse model by bovine serum albumin (BSA) injection to investigate whether hyperlipidemia could accelerate CKD progression and the underlying mechanisms. Finasteride was administrated to explore its potential therapeutic effects. The results of biochemical analyses and pathological examination showed that HFD-induced hyperlipidemia led to aggravated protein-overload nephropathy in mice along with an elevated level of circulating TMAO, which can be alleviated by finasteride treatment possibly through inhibition of Fmo3 in liver. The 16 S rRNA sequencing results indicated that HFD feeding altered the composition and distribution of gut microbiota in CKD mice contributing to the enhanced level of TMAO precursor TMA, while finasteride could exert beneficial effects *via* promoting the abundance of *Alistipes_senegalensis* and *Akkermansia_muciniphila*. Immunofluorescence staining (IF) and qRT-PCR results demonstrated the disruption of intestinal barrier by decreased expression of tight junction proteins including Claudin-1 and Zo-1 in HFD-fed CKD mice, which can be rescued by finasteride treatment. Cytokine arrays and redox status analyses revealed an upregulated inflammatory level and oxidative stress after HFD feeding in CKO mice, and finasteride-treatment could alleviate these lesions. To summarize, our study suggested that finasteride could alleviate HFD-associated deterioration of protein-overload nephropathy in mice by inhibition of TMAO synthesis and regulation of gut microbiota.

## Introduction

Chronic kidney disease (CKD) refers to abnormalities of kidney structure or kidney dysfunction lasting for more than 3 months (Stevens and Levin, 2013). The diagnosis criteria of CKD include GFR less than 60 ml/min/1.73 m^2^, albuminuria, abnormalities in urine test or kidney imaging examination, the renal tubular disorders, or with history of kidney transplantation (Chen TK et al., 2019). The occurrence of CKD is usually accompanied with obesity, which is prone to metabolic disorders and pro-inflammatory status (Eckardt et al., 2013; Reiss et al., 2015). It was reported that obesity has emerged as a leading cause of CKD, with recent estimations indicating that 24%–33% of all US CKD cases attribute to obesity (Wang et al., 2008). Since hyperlipidemia is the hallmark of obesity, we believe that it is of great importance to investigate the role of hyperlipemia in CKD progression.

Unhealthy diet especially high-fat diet (HFD) is the major cause of hyperlipidemia leading to CKD deterioration in patients. There has been accumulating evidences suggesting that individuals suffering from chronic kidney disease tend to develop microbial dysbiosis and gut barrier dysfunction (Anders et al., 2013; Knauf et al., 2019; Meijers et al., 2019). Gut microbes normally have a balanced relationship with their host and exert crucial effects on production of micronutrient as well as maintenance of immune homeostasis and energy metabolism, which can be disturbed by HFD. Thus, it is necessary to explore new therapeutic targets for intestinal dysfunction caused by HFD in CKD progression.

HFD can induce dysfunction of colonocyte, which escalates microbiota-derived trimethylamine N-oxide (TMAO) in circulation (Yoo et al., 2021). TMAO is a gut-derived uremic toxin, originating from choline, phosphatidyl choline (PC), L-carnitine, and certain marine fish. The gut microbiota converted these substances to generate TMA, which is then oxidized by flavin monooxygenase (FMO) enzyme in liver to produce TMAO (Janeiro et al., 2018). It has been proved that TMAO has a close relationship with inflammation, cardiovascular diseases, thrombosis and kidney diseases (Zeisel and Warrier, 2017; Abbasi, 2019; Yang et al., 2019; Liu et al., 2021). In our previous study, we reported that finasteride, a competitive and specific inhibitor of type II 5a-reductase, was able to downregulate the level of FMO3 (Liu et al., 2020). In addition, finasteride treatment can alter the gut microbiota composition (Diviccaro et al., 2019; Borgo et al., 2021). It also plays a protective role in kidney stones diseases (Sueksakit and Thongboonkerd, 2019), kidney transplantation (Antus et al., 2001), diabetic renal microvascular complications (Tian et al., 2015) and glioblastoma progression (Kim et al., 2021). However, whether finasteride is able to benefit HFD-associated CKD is still unclear. We proposed that finasteride could reduce serum TMAO and exhibit an HFD-protective effect in mice with proteinuric nephropathy. Hence, our present study aimed to investigate the possible relationship between hyperlipidemia and CKD progression, as well as whether there is a therapeutic effect of finasteride on HFD-associated CKD progression and its underlying mechanisms.

## Material and Methods

### Animal Experiments

**FIGURE 1:**
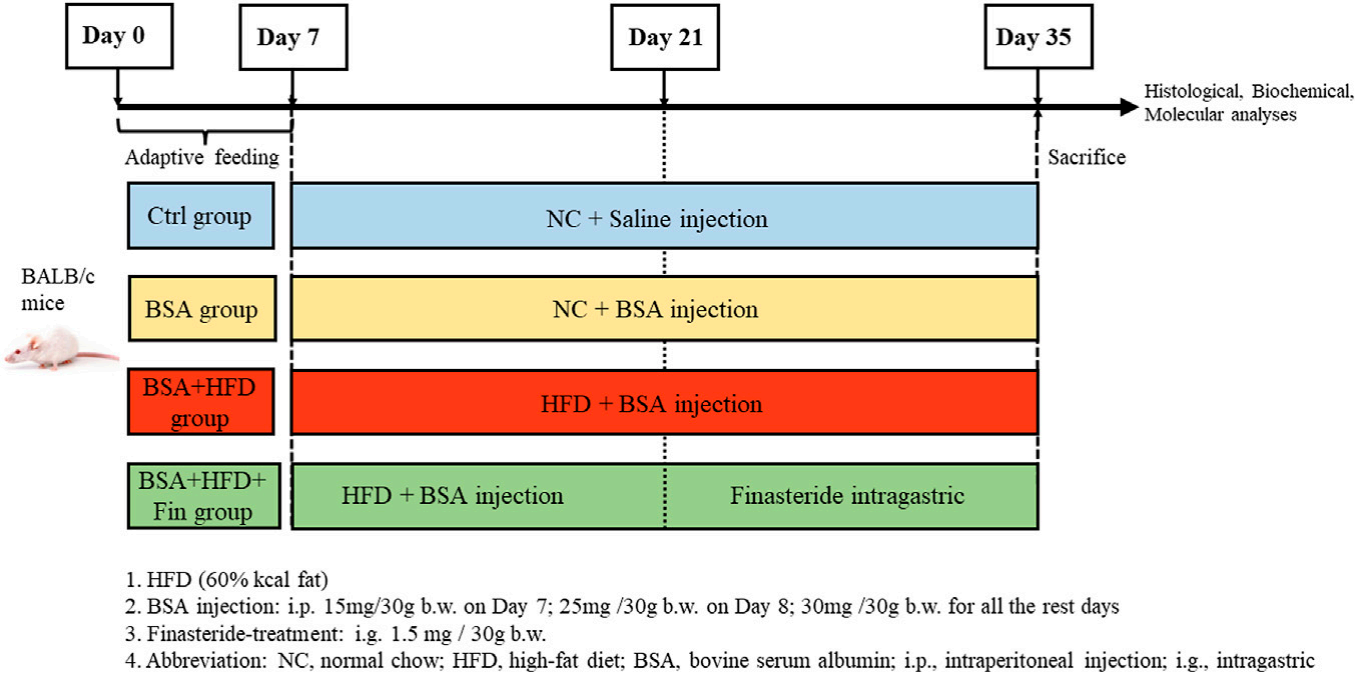
Experiment design.

Chronic kidney diseases (CKD) mouse models were generated with 10-week-old male Balb/c mice (Jihui labanimal, Shanghai, China) by bovine serum albumin (BSA) injection as previously described (Lai et al., 2021). All mice were housed in a constant-temperature room with a 12-h dark/12-h light cycle in a specific pathogen–free facility. To investigate the impact of high-fat diet (HFD) feeding on kidney functions in CKD mice, the mice were fed with HFD simultaneously with BSA injection for 4 weeks and treated with finasteride in the last 2 weeks. After 1 week of adaptive feeding, mice (*n* = 32) were randomly divided in 4 groups on Day 7 and subjected to the following treatment: 1) The control group (Ctrl group, *n* = 8): NC + equal volume of saline as BSA solution; 2) NC with BSA-injected group (BSA group, *n* = 8), NC + BSA i.p. injection at 30 mg/30 g body weight (with the dosage of 15 and 25 mg/30 g BSA on the first 2 days); 3) HFD with BSA-injected group (BSA + HFD group, *n* = 8): HFD + equal volume of BSA solution; 4) The Finasteride-treatment group (HFD + BSA + Fin group, *n* = 8): HFD + equal volume of BSA solution + finasteride at 1.5 mg/30 g body weight. (Figure 1).

The composition of HFD (D12492; FBSH Biotechnology Co., Ltd.) is 20% kcal protein, 20% kcal carbohydrate, and 60% kcal fat with 5.24 kcal/g; and NC feeding (LabDiet 5,053; LabDiet) consisted of 20.6% kcal protein, 67.4% kcal carbohydrate, and 12% kcal fat with 3.6 kcal/g for 4 weeks, respectively. All mice were sacrificed on Day 35. This study was approved by the Institutional Animal Care and Use Committee (IACUC) office of Fudan University (Approval Number: JS-315) (Derrien et al., 2004).

### Evaluation of Renal Function

Mouse fresh blood samples were collected immediately after sacrifice, and the supernatant was collected by centrifugation at 3,000 *g* for 10 min at 4°C and stored at −80°C until use. The levels of blood urea nitrogen (BUN), blood uric acid (UA) and serum creatinine (Scr) in serum were assessed by an automatic analyzer (ADVIA XPT, Siemens Healthcare Diagnostics Inc.). Urine samples were collected by metabolic cages, and the urine protein levels were measured with commercial kits (Nanjing Jiancheng Bioengineering Institute, China) according to the manufacturer’s instructions.

### Metabolite Measurements

Serum TMAO and TMA levels were measured using liquid chromatography coupled to tandem mass spectrometry (LC-MS/MS) (Zheng et al., 2021). Briefly, methanol was used to precipitate proteins (serum: methanol, 1:2, v/v). Then, the solution was well mixed by vortex, centrifuged at 14,000 g for 10 min at 4°C. After that, the supernatants were collected for analysis. Mass spectrometry experiments were performed on Triple TOF 5,600 + an orthogonal accelerated TOF MS (AB Sciex, United States) equipped with an electrospray ion source. 0.2% formic acid in methanol was applied to resolve the analytes. TMAO and TMA was assessed by electrospray ionization (ESI) in positive-ion mode with multiple reaction monitoring (MRM) of precursor and characteristic product ion transitions of m/z 76→58, and m/z 60→44. The parameters for the ion monitoring were optimized in individual mass spectrometers. Then Skyline software was used to normalize the original data and carry out further analysis.

### Histological and Immunofluorescence Analyses

Liver, kidney and colon tissues were fixed in 4% paraformaldehyde, routinely dehydrated, embedded and cut into sections of 4 μm thick. The tissue sections were stained with hematoxylin and eosin (H&E), oil red O, periodic acid Schiff (PAS) and Masson staining by kits (Servicebio, Wuhan, China) following manufacturer’s instructions. The percentage of mesangial hyperplasia was calculated as the ratio of the pink mesangial area and the total glomerular area in each glomerulus. The quantitative statistical method of collagen area from Masson staining: Collagen area fraction (%) = (MASSON staining collagen positive area/total area) * 100%”. Each group randomly selected four fields of vision, blinded by two researchers (magnification: 400×).

The colon tissue sections were routinely deparaffinized and antigen retrieval was performed with EDTA and blocked with 3%BSA. Primary antibodies including anti‐ZO‐1 (catalog no. 66452‐1; 1:100; ProteinTech Group) and anti-Claudin-1 (catalog no. 13050-1; 1:100; ProteinTech Group) primary antibody were applied for incubation at 4°C overnight followed by incubation of the corresponding secondary antibody (1:200) for an hour at room temperature, and then mounted with a medium containing DAPI for capturing pictures.

### The Quantitative Real-Time Reverse Transcription PCR (qRT-PCR) Assay

Liver and colon tissues were homogenized in TRIzol reagent (Thermo Fisher Scientific, Waltham) and total RNA were extracted according to the manufacturer’s instructions. Complementary DNA (cDNA) synthesis was performed using a commercial synthesis kit (EnzyArtisan, Shanghai, China). Quantitation of target gene mRNA was performed using S6 Universal SYBR qPCR mix (EnzyArtisan, Shanghai, China and an ABI PRISM 7900 HT Sequence Detection System (Applied Biosystems, Foster City, CA, Uinted States). Results were calculated and presented as relative expression of transcripts normalized to β-actin. Oligonucleotide primers were designed using NCBI Online Tools (https://www.ncbi.nlm.nih.gov/tools/primer-blast/). The primer sequences were as following: *Claudin-1* Forward: 5′‐TGC​CCC​AGT​GGA​AGA​TTT​ACT‐3′ and *Claudin-1* Reverse: 5′‐CTT​TGC​GAA​ACG​CAG​GAC​AT‐3′. *Zo-1* Forward: 5′‐GCT​TTA​GCG​AAC​AGA​AGG​AGC‐3′ and *Zo-1* Reverse: 5′‐TTC​ATT​TTT​CCG​AGA​CTT​CAC​CA‐3′. *Fmo3* Forward: 5′‐CAG​GAA​TAT​GGA​AGG​GAA​AAC​G‐3′ and *Fmo3* Reverse: 5′‐CGA​CTC​ATC​ACC​CAA​GAA​CCA​C‐3′. *Fxr* Forward: 5′‐GGG​GAT​GAG​CTG​TGT​GTT​GTC​T‐3′ and *Fxr* Reverse: 5′‐GGC​GTT​CTT​GGT​AAT​GCT​TCT​TC‐3′. *Cyp7a1* Forward: 5′‐CAC​CAT​TCC​TGC​AAC​CTT​CTG​G‐3′ and *Cyp7a1* Reverse: 5′‐ATG​GCA​TTC​CCT​CCA​GAG​CTG​A‐3′. *Pkm* Forward: 5′‐CAG​AGA​AGG​TCT​TCC​TGG​CTC​A‐3′ and *Pkm* Reverse: 5′‐GCC​ACA​TCA​CTG​CCT​TCA​GCA​C‐3′. *Nrf2* Forward: 5′‐CAG​CAT​AGA​GCA​GGA​CAT​GGA​G‐3′ and *Nrf2* Reverse: 5′‐GAA​CAG​CGG​TAG​TAT​CAG​CCA​G‐3′. *β-actin* Forward: 5′‐CAT​TGC​TGA​CAG​GAT​GCA​GAA​GG‐3′ and *β-actin* Reverse: 5′‐TGC​TGG​AAG​GTG​GAC​AGT​GAG​G‐3′.

### Lipid Analysis

Concentrations of Triglyceride (TG), total cholesterol (TG), low-density lipoprotein cholesterol (LDL), and high-density lipoprotein cholesterol (HDL) in serum were measured with commercial kits (Nanjing Jiancheng Bioengineering Institute, China) according to the manufacturer’s instructions

### Redox Status Parameters

Renal superoxide dismutase (SOD) activity was assessed using the commercial colorimetric kit supplied by (catalog no. A-001-3, Nanjing Jiancheng Bioengineering Institute, China). This assay relies on the xanthine oxidase reaction system to produce superoxide anion radicals (O_2_^−^). O_2_^−^·can oxidize hydroxylamine to form nitrite, which can turn purplish red under the action of chromogenic agent, and SOD can inhibit O_2_^−^ and produce less nitrite.

Serum glutathione (GSH) level was measured using the commercial kit supplied by (catalog no. A-061-1, Nanjing Jiancheng Bioengineering Institute, China) following manufacturer’s instructions. In this assay, the contents of total and oxidized glutathione were determined by DTNB cyclic reaction.

Renal malondialdehyde (MDA) level was measured using the commercial kit supplied by (catalog no. A-003-1, Nanjing Jiancheng Bioengineering Institute, China) following manufacturer’s instructions. In this assay, malondialdehyde (MDA) can combine with thiobarbituric acid (TBA) to form a red product with a maximum absorption peak at 532 nm.

### Cytokine Array

Mouse cytokine array (catalog no. ab133993, Abcam) designed to detect the 22 cytokines was performed following the manufacturer’s instructions. Briefly, the serum of mouse was diluted by five times and then incubated with the membrane at 4°C overnight, the analysis was carried out by chemiluminescent western blot assay, using biotinylated detector antibodies and streptavidin HRP. Targets of this ELISA-like array are granulocyte-colony stimulating factor (GCSF), granulocyte-macrophage colony stimulating factor (GM-CSF), IL-2/3/4/5/6/9/10/12p40/p70, 12p70, 13, 17, interferon gamma (IFN-γ), monocyte chemoattractant protein 1 and 5 (MCP-1 and MCP-5), regulated on activation normal T cell expressed and secreted (RANTES), stem cell factor (SCF), soluble tumor necrosis receptor factor 1 (sTNFR1), tumor necrosis factor alpha (TNF-α), thrombopoietin, and vascular endothelial growth factor (VEGF). Results were analyzed with ImageJ software as previously described (Huante-Mendoza et al., 2016).

### Gut Microbiota Analysis

According to the previous study, fresh fecal samples were collected during the final 5 days for the gut microbial analysis. Bacterial genomic DNA was extracted from frozen fecal samples stored at −80°C with a QIAamp Fast DNA stool Mini Kit (catalog no. 51604, Qiagen). The variable region 3–4 (V3-V4) of the 16 S rRNA gene comprising were amplified by qRT-PCR using specific bacterial primers (F primer: 5′-ACT​CCT​ACG​GGA​GGC​AGC​A-3′; R primer: 5′-GGACTACHVGGGTWTCTAAT-3′) (Chen K et al., 2019). High-throughput pyrosequencing of the qRT-PCR products was performed on an Illumina MiSeq platform at Biomarker Technologies Co., Ltd. (China). Construction of sequencing libraries and paired-end sequencing was performed on an Illumina NovaSeg6000 platform at Biomarker Technologies Co., Ltd. (Beijing, China) according to standard protocols. Paired-end reads were merged using FLASH v1.2.7, and tags with more than six mismatches were discarded. The raw paired-end reads were merged using FLASH v1.2.7 (Chen K et al., 2019), and tags with more than six mismatches were discarded, according to the unique barcodes. The merged tags with an average quality score <20 in a 50 bp sliding window were determined using Trimmomatic (Sheng et al., 2019) and those shorter than 350 bp were removed. Possible chimeras were further removed and the denoised sequences were clustered into operational taxonomic units (OTUs) with 97% similarity using USEARCH (version 10.0). Taxonomy was assigned to all OTUs by searching against the Silva databases (Release128) using QIIME software. Raw sequences were deposited in the Sequence Read Archive database (http//wwwncbinlm.nih.gov/sra), with the accession numbers ranging from SAMN* to SAMN*. The alpha diversity index was evaluated using Mothur software (version, v.1.30). To compare the diversity index among samples, the number of sequences contained in each sample was standardized. Analysis treasure included OTU rank, rarefaction, and Shannon curves, and the Shannon, Chao1, and ACE indexes were calculated. For beta diversity analysis, heatmaps of RDA-identified key OTUs, PcoA, NMDS (Looft et al., 2012), and UPGMA were obtained using QIIME. The LDA-effect size (LEfSe) method was used for the quantitative analysis of biomarkers in each group. Briefly, LEfSe analysis, an LDA threshold >4, the non-parametric factorial Kruskal–Wallis sum-rank test, and the unpaired Wilcoxon rank-sum test were performed to identify the most differently abundant taxa (Segata et al., 2011; Parks et al., 2014).

### Statistical Analysis

Results are presented as mean ± Standard deviation (SD). At least four sets of repeated experiments were performed and n-values indicate the number of animals analyzed in each group. All calculations, curve fitting and statistical analyses were performed with GraphPad Prism (version 7.03; GraphPad Software, La Jolla, CA, United States). Statistical significances between different data sets were analyzed using one-way ANOVA followed by the Bonferroni post-hoc test. Differences were considered significant at an error probability of less than 0.05.

## Results

### High-Fat Diet Treatment Led to Deteriorated Protein-Overload Nephropathy in Mice, Which can Be Alleviated by Finasteride

**FIGURE 2:**
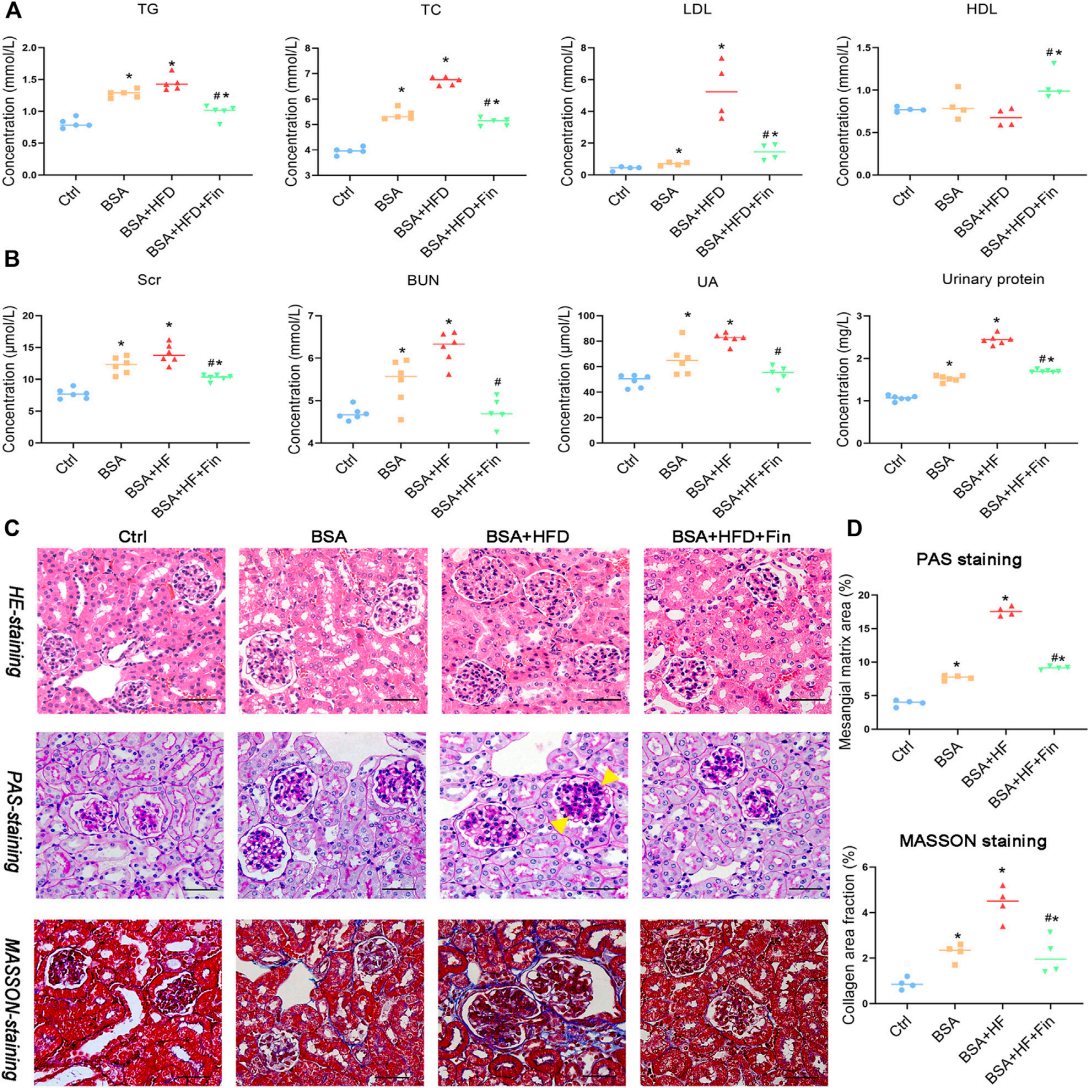
Finasteride alleviated the high-fat diet (HFD) induced hyperlipidemia and renal injury. Urine samples of four groups of mice were collected by metabolic cage before sacrifice for evaluation of urinary protein. Serum samples were collected after sacrifice for evaluation of lipid profiles and renal injury markers. **(A)** Concentrations of Triglyceride (TG), total cholesterol (TG), low-density lipoprotein-Cholesterol (LDL), and high-density lipoprotein-Cholesterol (HDL) in serum were measured for comparison. **(B)** Concentrations of serum creatinine (Scr), blood urea nitrogen (BUN) and uric acid (UA) in serum and protein in urine were assessed. **(C)** Kidney sections were stained with Hematoxylin-eosin staining (C, upper), Periodic acid-Schiff staining (C, middle) and Masson’s Trichrome staining (C, bottom). The yellow arrow indicated the mesangial matrix expansion sites. Scale bars: 100 μm. **(D)** Analyses of mesangial matrix area and collagen area fraction of kidney tissues in Ctrl, BSA, BSA + HFD, and BSA + HFD + Fin groups of mice were performed for comparison. All data were expressed as the mean ± SD (*n* = 4–6) and subjected to one-way ANOVA followed by the Bonferroni *post hoc* test. ^*^ *p* < 0.05 vs. Ctrl group, ^#^ *p* < 0.05 vs BSA + HFD group.

The increased urinary protein level is one of the signature characteristics of chronic kidney disease (CKD). Therefore, in this study, protein-overload nephropathy mouse model was generated by intraperitoneal (i.p.) injection. To investigate whether finasteride could rescue hyperlipidemia-promoted progression of CKD, mice were simultaneously treated with 60% high-fat diet (HFD) with or without finasteride for the last 2 weeks as comparison (Figure 1). As a result, HFD treatment led to significant hyperlipidemia, reflected by significantly upregulated levels of total cholesterol (TC), triglyceride (TG), and low-density lipoprotein cholesterol (LDL) and downregulated high-density lipoprotein cholesterol (HDL) level in HFD with BSA injection group (BSA + HFD group) when compared to the normal chow control group (Ctrl group) and normal chow with BSA injection group (BSA group), while the lipid abnormalities could be rescued by finasteride (BSA + HFD + Fin group) (Figure 2A). Meanwhile, BSA injection led to obvious renal injury reflected by the significantly elevated levels of urinary protein, serum creatinine, urea and uric acid when compared to Ctrl group mice (Figure 2B). The pathological conditions of kidneys of four groups of mice were also analyzed by section staining including hemoxylin-eosin (HE) staining, periodic acid-Schiff (PAS) staining and MASSON staining (Figures 2C,D). The results indicated that the kidneys of BSA group mice reflected a significant acceleration of renal mesangial proliferation and increase of glomerular collagen deposition than the Ctrl group. HFD feeding further exacerbated these CKD symptoms, while finasteride treatment effectively alleviated them (Figure 2).

### Finasteride Rescued the Abnormally Enhanced Level of Trimethylamine N-Oxide in High-Fat Diet-Fed Chronic Kidney Diseases Mice

**FIGURE 3:**
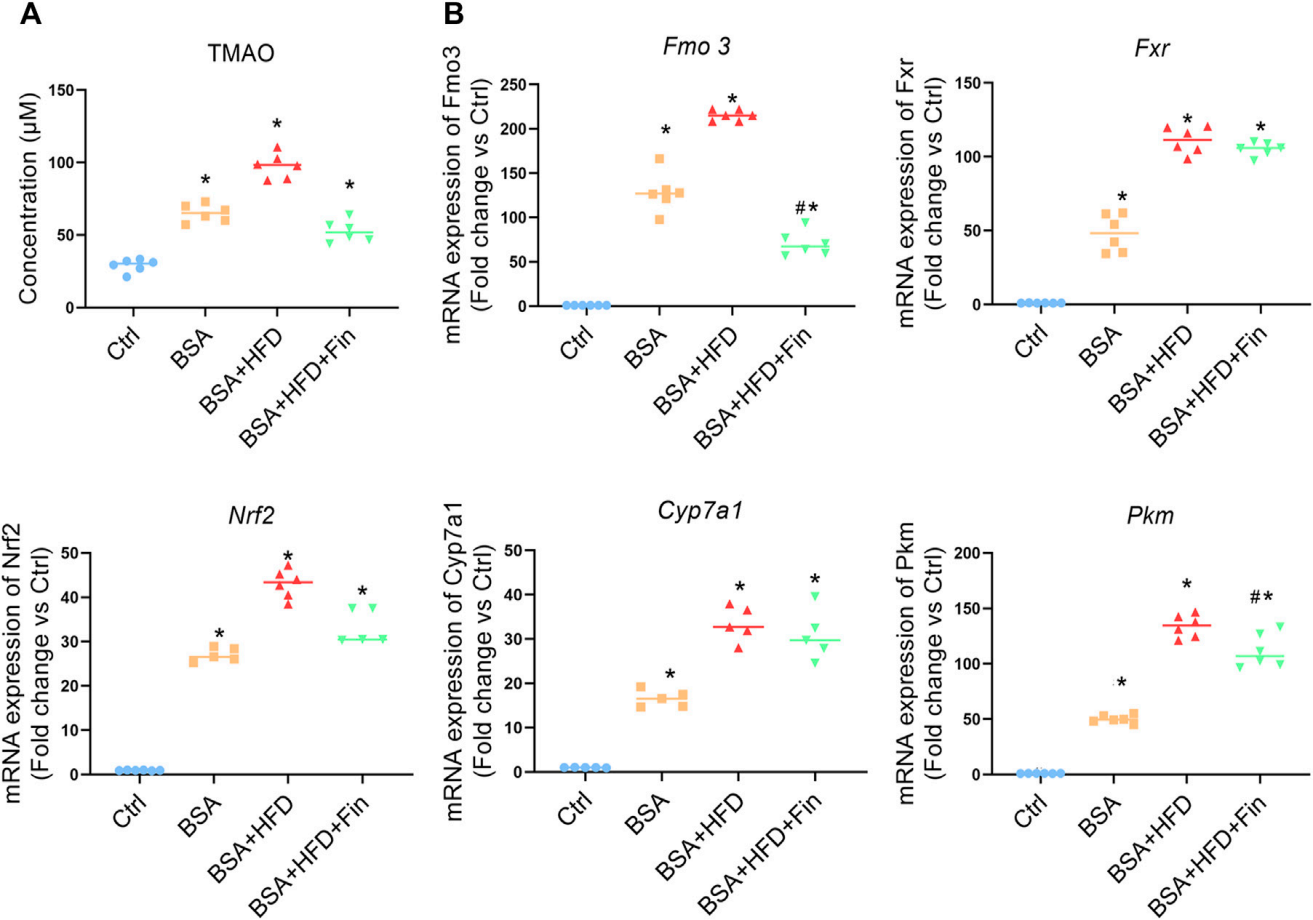
Finasteride reduced TMAO concentration and regulated transcriptional disturbance of metabolic genes caused by HFD. Serum and liver samples of four group of mice were obtained after sacrifice. **(A)** The levels of the TMAO in the serum of mice in each group were measured using LC-MS. **(B)** qRT-PCR analyses of hepatic metabolic genes in four groups of mice were performed. *Fmo3*: the flavin monooxidase 3. *Fxr*: farnesoid x receptor. *Cyp7a1*: cholesterol 7α-hydroxylase. *Nrf2*: Subcellular localization of nuclear factor E2-related factor 2. *Pkm*: pyruvate kinase M type. After normalization against β-actin, the relative abundance of *Fmo3, Fxr, Cyp7a1, Nrf2*, and *Pkm* mRNA levels were calculated and presented as fold changes. All data were expressed as the mean ± SD (*n* = 4–6) and subjected to one-way ANOVA followed by the Bonferroni *post hoc* test. ^*^ *p* < 0.05 vs. Ctrl group, ^#^ *p* < 0.05 vs. BSA + HFD group.

As is known, 4-weeks HFD feeding is inadequate to induce neither severe organ damage nor dysfunction, which is reflected by the staining results of liver sections (Supplementary Figure S1) (van der Heijden et al., 2015; Zhang et al., 2020). The previous study of our research group demonstrated that finasteride could rescue the elevation of TMAO caused by HFD feeding in mice (Liu et al., 2020). According to the LC-MS results, the TMAO concentration is significantly enhanced after BSA injection, and further elevated when HFD is simultaneously fed, while finasteride treatment could lower the TMAO to the similar level of BSA group (Figure 3A, Supplementary Figure S2). Fatty acid is metabolized in liver, through various metabolic enzymes. HFD-feeding may lead to a transcriptional disturbance of metabolic genes in liver. Therefore, we evaluated the mRNA level of several hepatic enzymes including flavin monooxidase 3 (*Fmo3*), farnesoid x receptor (*Fxr*), subcellular localization of nuclear factor E2-related factor 2 (*Nrf2*), cholesterol 7α-hydroxylase (*Cyp7a1*), and pyruvate kinase M type (*Pkm*), all of which have been implicated in metabolic disturbances (Kübeck et al., 2016; Xie et al., 2016; Hu Q et al., 2021) (Figure 3B). Here, we noted that finasteride is able to downregulate the *Fmo3* expression, while FMOxs is the enzyme responsible for TMAO generation (Steinke et al., 2020). With the similar change pattern of circulating TMAO, *Fmo3* expression and renal injury level, we suggested that the capability of finasteride to reduce the *Fmo3* expression and TMAO level is highly related to the improved renal function in HFD-fed CKD mice.

### High-Fat Diet Feeding and Treatment of Finasteride Altered the Composition and Distribution of Gut Microbiota in Chronic Kidney Diseases Mice

**FIGURE 4:**
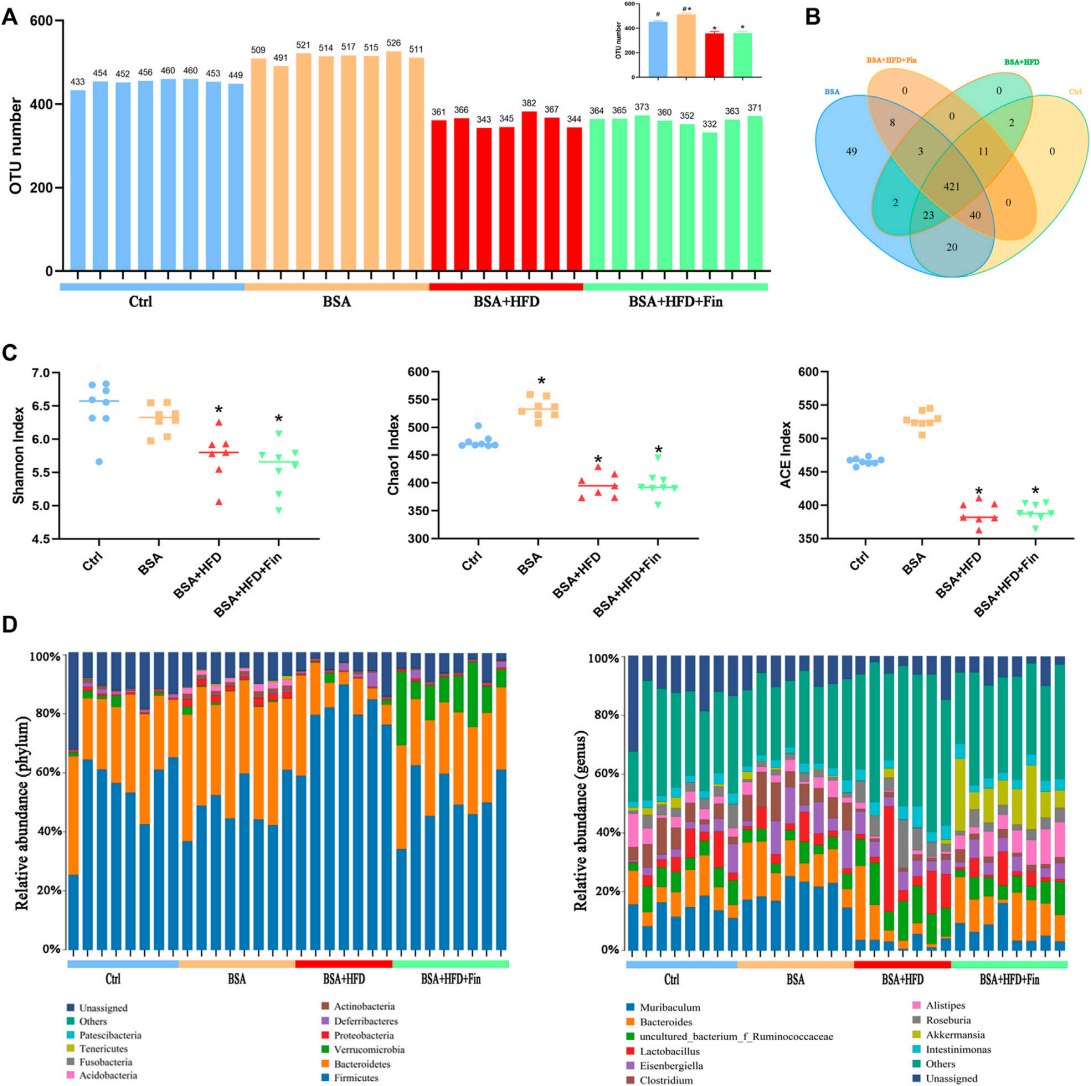
HFD lead to gut microbiota dysfunction in CKD mice, which could be alleviated by finasteride treatment.Fecal samples of four groups of mice were collected for analysis of operational taxonomic units and taxonomic distribution of gut microbiota. **(A)** Distribution of the number of OTUs in each sample. **(B)** Venn diagram of operational taxonomic units. **(C)** Alpha diversity indices of OTU distribution in each sample. **(D)** Taxonomic distribution of gut microbiota in the mice of each group at the phylum and genus level. All data were expressed as the mean ± SD and subjected to one-way ANOVA followed by the Bonferroni *post hoc* test. ^*^ *p* < 0.05 vs. Ctrl group, ^#^ *p* < 0.05 vs. BSA + HFD group.

To determine the effect of HFD feeding with or without finasteride treatment on the mouse gut microbiota, high-throughput sequencing of 16 S rRNA in the cecal content was performed. Feces of mice from the Ctrl, BSA, BSA + HFD, and BSA + HFD + Fin groups were collected to assess the specific composition and distribution of gut microbiota by sequencing 16 S rRNA V3 + V4 region of the bacteria. A total of 2,413,991 pairs of reads were obtained by sequencing 37 samples. After filtering, a total of 2,406,345 clean reads were generated with at least 55,304 clean reads per sample. An average of 77,624 clean reads were generated (Supplementary Table S1). QIIME (Version 1.8.0) UCLUST software was used to cluster the tags into Operational Taxonomic units (OTU) based on 97% sequence similarity. The number of OTUs in these four groups showed a significant difference and there was an increase trend between the mean OTUs in the BSA + HFD group to the BSA + HFD + Fin group (Figure 4A). Then, we generated a Venn diagram of OTUs (Figure 4B), where the results showed that the four groups had 427 operational taxonomic units overlapping but no unique OUT after finasteride treatment. Afterwards, we compared the α-diversity among these four groups (Figure 4C, Supplementary Table S2). Consistent with the change in the number of OTUs, HFD could reduce the richness and diversity of gut microbiota while finasteride may have some ameliorative effects.

**FIGURE 5:**
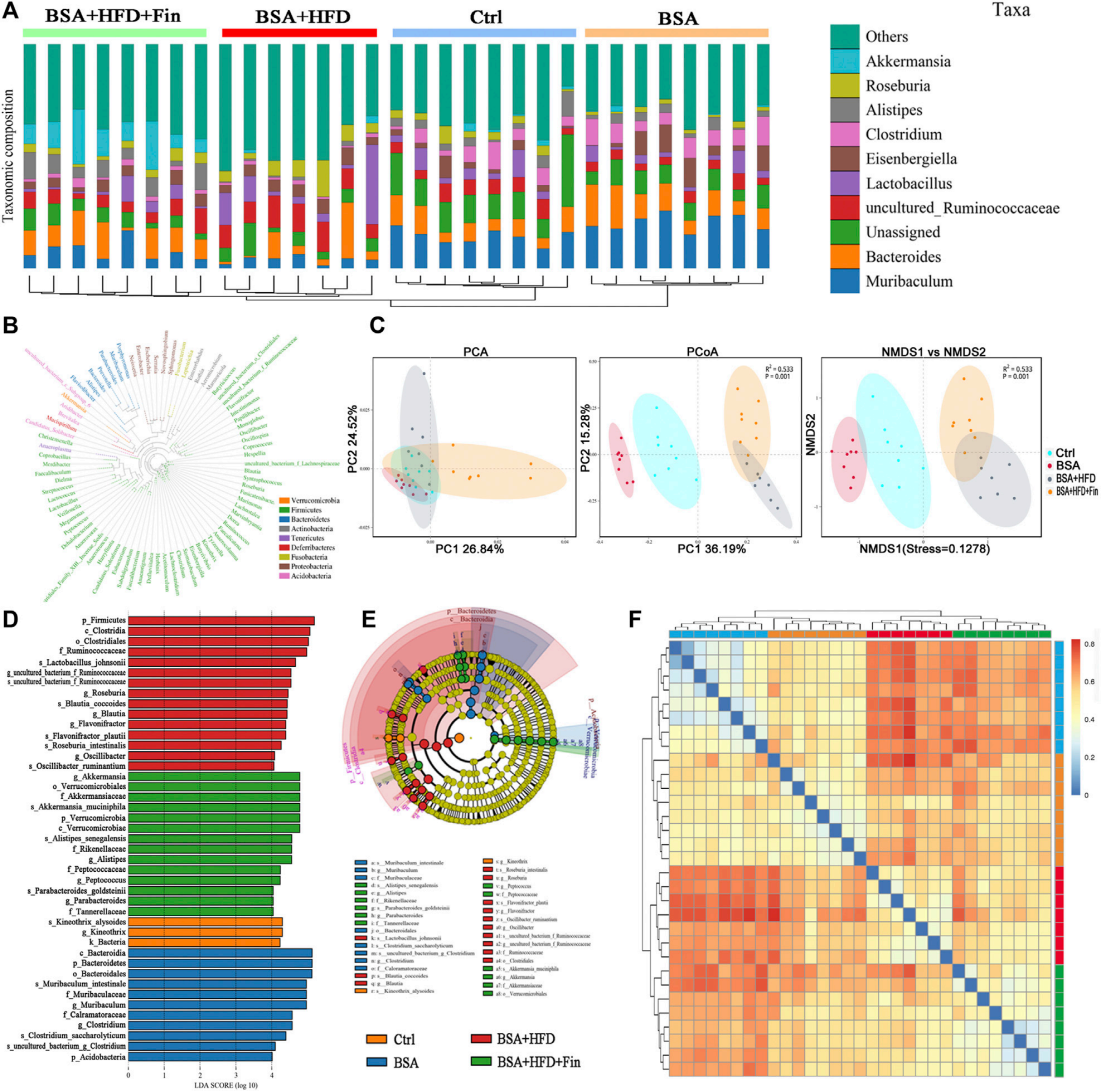
Specific microbiome populations in each group of mice. **(A)** Combined UPGMA clustering tree and histograms of the gut microbiota of the mice in each group. **(B)** Phylogenetic tree based on the OTUs in each sample at the genus level. **(C)** Beta diversity analysis of the OTU distribution in each sample. **(D)** Heatmap analysis on the gut microbiota of mice in each group. **(E) (F)** Line discriminant analysis (LDA) effect size analysis of the gut microbiota in each sample.

Each OTU can be assigned as a species by comparing the sequence of the operational taxonomic units with the microbial reference database. Meanwhile, the community composition of each sample was also calculated. The QIIME software was used to generate the abundance tables for each taxonomic group at the different classification levels (phylum, class, order, family, genus, and species) (Figure 4D, Supplementary Table S3). Hierarchical cluster analysis by unweighted pair-group method with arithmetic mean (UPGMA) suggested that there were no homology and close genetic background between the gut microbiota of these four groups mice. A diagram based on the UPGMA clustering tree and histograms is shown in Figure 5A. Phylum level analysis showed that after finasteride treatment, the level of *Verrucomicrobia* was increased significantly, and in the BSA + HFD group, the abundance of *Bacteroidetes* was decreased notably, while the level of *Firmicutes* increased. Conversely, in the other three groups, the ratios of *Bacteroides* to *Firmicutes* were all higher than the BSA + HFD group, which indicated a better gut microbiota environment (Ley et al., 2006). Species level analysis showed that compared to the BSA + HFD group, finasteride-treatment could upregulate the abundance of *Akkermansia_muciniphila* and *Alistipes_senegalensis,* whereas reduce the abundance of *uncultured_Ruminococcaceae*. It was also shown that the diversity of gut microbes in the mice of each group was mostly attributable to microbes from the phylum *Firmicutes* (Figure 5B). β-diversity analysis was produced mainly by the Bray-Curtis Algorithm with principal coordinates analysis (PCOA), principal component analysis (PCA), and non-metric multi-dimensional analysis scaling (NMDS), which could be used to analyze the differences between these four groups of microbes (Figure 5C). The analysis results showed that there were significant differences in the distribution of microbial communities between these four groups (*p* = 0.001). To further understand the similarity among each sample, we produced a heatmap analysis on the gut microbiota of mice in these groups. It showed that there was little difference between the BSA and Ctrl group, but a great gap from BSA + HFD group to Ctrl group, and finasteride-treatment may adjust this distinction (Figure 5D). In addition, Line Discriminant Analysis (LDA) Effect Size (LEfSe) was used to identify high-dimensional biomarkers in each group of gut microbiota. The LDA score was set as 4.0, and an LDA score greater than 4 is considered as an important biomarker for different species. As LDA score distribution result and Cladogram analysis showed, in the BSA + HFD + Fin group, *g_Akkermansia*, *o_Verrucomicrobiales*, *S_Akkermansia_mucipiphila* family of microbes were the predominate microorganisms. On the other side, the microbes belonging to *p_Firmicutes*, *c_Clostridia*, *o_Clostridiales*, *F_Ruminococcaceae* were the dominant microorganisms in the BSA + HFD group (Figures 5E,F). Kyoto Encyclopedia of Genes and Genomes (KEGG) metabolic pathway analysis of those changed signaling pathways were showed in the Supplementary Figure S3.

We also measured serum TMA level, the precursor of TMAO, of the four groups of mice by LC-MS (Supplementary Figure S4). The HFD feeding resulted in changes in gut microbiota and increased levels of TMA. These results illustrated that HFD-feeding led to the enhanced abundance of bacteria that produce TMA, while finasteride reduced TMA level and lowered the abundance of some bacteria which may be harmful to health.

### High-Fat Diet Feeding Disrupted Gut Barrier as Well as Promoted Inflammation and Oxidative Stress in Chronic Kidney Diseases Mice, Which Can Be Rescued by Finasteride Treatment

**FIGURE 6:**
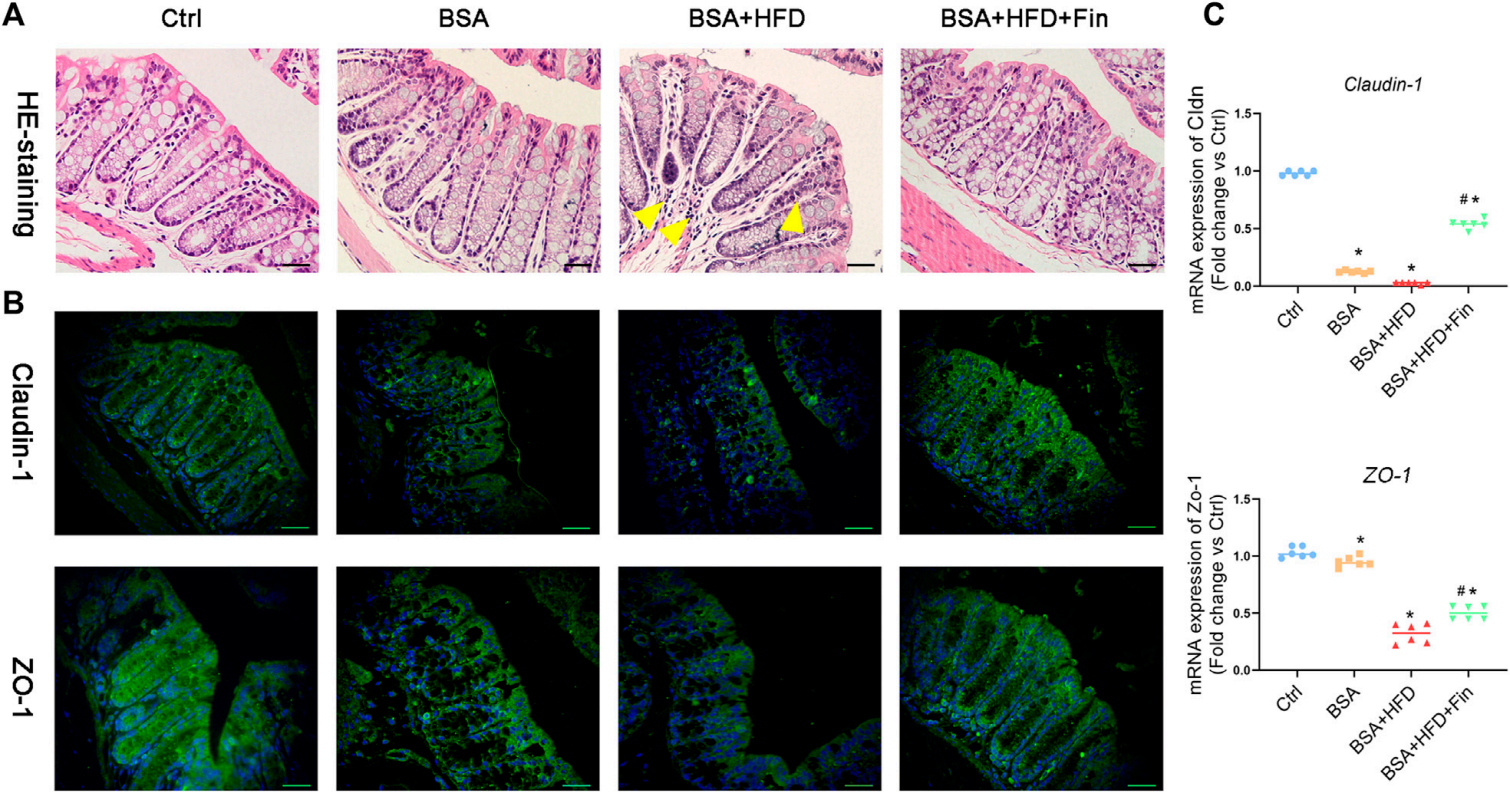
Finasteride alleviated inflammation and restored intestinal tight junction by reducing TMAO level. After sacrifice, colon tissues were collected for paraffin section preparation. **(A)** Colon sections were stained with Hematoxylin-eosin staining. The yellow arrow indicated the inflammatory cell infiltration in the BSA + HFD group. **(B)** Immunofluorescence staining of Claudin-1 and Zo-1 were performed. Scale bar: 50 μm. **(C)** qRT-PCR analyses of *Claudin-1* and *Zo-1* mRNA expression in the colon tissue were performed. After normalization against β-actin, the relative abundance of *Claudin-1* and *Zo-1*mRNA levels were calculated and presented as fold changes. All data were expressed as the mean ± SD (*n* = 6) and subjected to one-way ANOVA followed by the Bonferroni *post hoc* test. ^*^ *p* < 0.05 vs. Ctrl group, ^#^ *p* < 0.05 vs. BSA + HFD group.

To further investigate the possible mechanisms underlying gut-kidney interactions in the condition of HFD feeding, we also look into more phenotypes including the gut barrier permeability and inflammation status. Firstly, we probed whether there were differences in gut barrier disruptions among these groups by histological staining of colon tissues. The H&E staining results indicated more inflammatory cell infiltration in the BSA + HFD group compared to the Ctrl group (Figure 6A). Moreover, we noticed the obvious color changes of goblet cells from light purple to deep and deeper purple in the Ctrl, BSA and BSA + HFD group, indicating changes in the composition and pH value of mucus, which could be reversed by finasteride administration. On the other hand, tight junction proteins, including Claudin-1 and ZO-1, serve as the basic composition of the paracellular permeability barrier (Zeisel et al., 2019). In order to observe the regulatory effect of finasteride on intestinal tight junction protein more intuitively, immunofluorescence staining for Claudin-1 and ZO-1 protein in colon tissues were performed (Figure 6B). Compared with the Ctrl group, the BSA + HFD group presented a considerable loss in the contents of Claudin-1 and ZO-1 protein. Notably, the mice with lower serum concentration of TMAO by finasteride showed a recovery in the contents of these proteins. Consistently, these results were also confirmed by qRT-PCR (Figure 6C).

**FIGURE 7:**
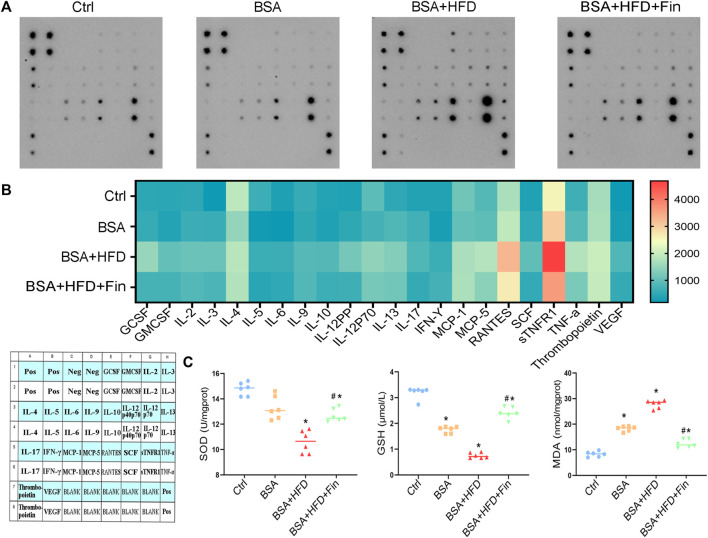
Finasteride treatment results in down-regulated pro-inflammatory level and less oxidative stress in mice. Serum samples were collected from four groups of mice after sacrifice for analyses of cytokine membrane array and oxidative stress evaluation. **(A)** The result of cytokine membranes is captured. **(B)** A heatmap of the expression levels of cytokines in these four groups and a cytokine membranes array map were generated according to the cytokine array results. **(C)** Levels of superoxide dismutase (SOD), glutathione (GSH) and malondialdehyde (MDA) in serum were measured. All data were expressed as the mean ± SD (*n* = 4–6) and subjected to one-way ANOVA followed by the Bonferroni *post hoc* test. ^*^ *p* < 0.05 vs. Ctrl group, ^#^ *p* < 0.05 vs. BSA + HFD group.

It is reported that abnormally enhanced TMAO resulted in pro-inflammatory status (Dabke et al., 2019), thus we measured the expression of 22 cytokines in serum using a cytokine membrane array. The results demonstrated that the expression of inflammatory factors was broadly increased in the BSA + HFD group as compared to BSA group, including *GCSF*, *IL-12*, *MCP-5*, *RANTES*, *sTNFR1*, *TNF-α*, and *VEGF*, while finasteride treatment could remarkably downregulate these inflammatory factors (Figures 7A,B). Last but not the least, we evaluated the kidney oxidative stress levels in the mice of these four groups. HFD treatment further exacerbate BSA injection-induced decrease in the levels of serum glutathione (GSH) and renal superoxide dismutase (SOD), and administration of finasteride effectively rescued them (Figure 7C). The levels of malondialdehyde (MDA) in serum were also assessed. Finasteride significantly reversed the enhanced MDA in BSA + HFD group to the similar level as BSA group (Figure 6E). These evidences showed that HFD feeding led to higher levels of inflammation and lower levels of antioxidant capacity in CKD mice. By incorporating all the results, we speculated that the hyperlipidemia may play an important role in deterioration of CKD, while inhibiting the elevation of TMAO level can alleviate the adverse effects of hyperlipidemia.

## Discussion

Excessive intake of dietary fat leads to abnormal metabolite deposition with potential to develop various chronic diseases, especially kidney diseases (Rohr et al., 2020; Tung et al., 2021). Severe hyperlipidemia could trigger the activation of pro-inflammatory, pro-fibrogenic, and pro-apoptotic signaling pathways, causing cellular damage cascade (Yamamoto et al., 2017; Sun et al., 2020). In this study, we generated a BSA-induced protein-overload nephropathy mouse model and suggested TMAO as a potential therapeutic target thus finasteride as a potent drug for alleviation of HFD-associated chronic renal injury.

To investigate whether hyperlipidemia can accelerate CKD progression, we fed the CKD mice with HFD simultaneously for 4 weeks (Figure 1). The deterioration of renal functions was observed reflected by the intensification of proteinuria, destruction of renal structure (Figure 2) and elevation of renal microinflammation (Figure 2), which can be alleviated by finasteride treatment. Finasteride, which originally aimed to treat prostate hyperplasia and androgenetic alopecia, could reduce TMAO level in HFD-fed mice by inhibiting the FMO3, a key enzyme involved in the process of TMA-to-TMAO conversion (Liu et al., 2020). Our previous clinical study demonstrated that the elevation of TMAO was associated with the progression of CKD (Steinke et al., 2020). In this regard, we examined the circulating level of TMAO in all groups of mice. The results indicted the positive correlation between TMAO level and severity of renal dysfunction (Figures 2, 3). Meanwhile, we detected HFD-induced abnormal alterations of liver metabolic enzymes, especially a significant increase of FMO3 which is consistent with the increase of circulating TMAO (Figure 3). Here, finasteride demonstrated its efficacy in prevention of CKD progression possibly through inhibition of FMO3 thus TMAO synthesis.

To further analyze the therapeutic impact of finasteride on HFD feeding-induced dysfunction of gut microbiota, we conducted 16s rRNA sequencing. The analytical results confirmed that HFD could induce energy metabolism disorder of colonic epithelial cells and increase *Enterobacteriaceae* proportion to escalate TMA level (Yoo et al., 2021). In our present study, the proportion of *Enterobacteriaceae* was higher in the BSA + HFD group (0.21% ± 0.04%) and BSA + HFD + Fin groups (0.21% ± 0.06%) than that in the Ctrl group (0.15% ± 0.05%), suggesting that HFD feeding can indeed elevate TMA level. However, as the proportion of *Enterobacteriaceae* was similar, we speculated that finasteride might reduce TMA content by affecting other bacteria. Previous studies have reported that *uncultured_Ruminococcaceae* can increase TMA-production, while *Bacteroides* and *Akkermansia* can reduce the concentration of TMA (Chen et al., 2016). Consistently, we found that finasteride-treatment could upregulate the abundance of *Akkermansia_muciniphila (A. muciniphila)* and *Bacteroides* and reduce the abundance of *uncultured_Ruminococcaceae* (Figure 4 *).* Interestingly, *A. muciniphila,* a genus of commensals in the *Verrucomicrobia* phylum (Derrien et al., 2010), is a mucin-consuming bacterium that may play an important role in maintaining integrity of the intestinal mucosal barrier and has anti-inflammatory properties (Chelakkot et al., 2018). Mounting evidences showed that the decreased level of *A. muciniphila* was considered to be related to the onset of various diseases (Zhai et al., 2019),especially metabolic disorders and inflammatory diseases, including obesity, type 2 diabetes, inflammatory bowel disease (IBD), autism and atopy (Everard et al., 2013; Grander et al., 2018). Besides, daily administration of live *A. muciniphila* grown on a mucus-based medium can counteract the development of HFD-induced obesity and gut barrier dysfunction. Plovier et al. propounded that Amuc_1,100, a specific protein isolated from the outer membrane of *A. muciniphila,* could upregulate intestinal tight junctions and reduce high-lipid-mediated endotoxemia (Plovier et al., 2017). Meanwhile, the study of Li et al. (2016) demonstrated that *A. muciniphila* -mediated reduction of TNF-α and MCP-1 levels could be attributed to the induction of intestinal expression of the tight junction proteins, in order to reverse the damage to the gut barrier caused by an HFD which was consistent with our present result (Figure 7). Another species of our great interest is *Alistipes_senegalensis.* Studies have confirmed that it is negatively correlated with the progression of chronic liver failure (Wang et al., 2021) and it was also significantly reduced in the gut microorganisms of Crohn’s disease patients (Hu et al., 2021), which may suggest that *Alistipes_senegalensis* plays a protective role in chronic kidney disease. These results suggested that finasteride may play a renal protective role by positively modulating beneficial intestinal microbiome.

The results of the alteration of gut microbiota are consistent with that in intestinal tight junctions. It is well known that HFD can disrupt the intestinal tight junction (Cremonini et al., 2019; Shi et al., 2019), thus we investigated whether finasteride treatment could ameliorate these pathological changes. According to IF and qRT-PCR results, we found that the expression of gut tight junction markers including Claudin-1 and ZO-1 in BSA + HFD mice are significantly lower than those in the Ctrl and BSA group, while finasteride-treatment promisingly restored it (Figure 6). As the disrupted intestinal barrier may promote the release of inflammatory factors and trigger oxidative stress, we then examined if finasteride treatment-associated TMAO decline can rescue the pro-inflammatory state. The results showed that the expression of pro-inflammatory factors including *GCSF*, *IL-12*, *MCP-5*, *RANTES*, *sTNFR1*, *TNF-α*, and *VEGF* was broadly increased in the BSA + HFD group, while treatment of finasteride could remarkably downregulate it as well as restore the antioxidant capacity. Among them, IL-12, TNF-α, and VEGF have been shown to be associated with intestinal microbiota disruption in CKD (Ji et al., 2021; Margiotta et al., 2021). Il-12 was significantly increased in the model of obstructive nephropathy with increased infiltration of macrophages (Lu et al., 2018). Mice with deficiency of TNFα exhibited decreased glomerular and tubular damage and attenuated kidney fibrosis in the models (Wen et al., 2019). It was also reported that VEGF level was positively correlated with Scr level in mice with acute kidney injury (Qin et al., 2020). All these above evidences reach to a plausible conjecture that inflammation may play a key role in the gut microbiota-kidney regulatory axis.

Nevertheless, our study has some limitations. The specific effect of finasteride on *A. muciniphila* is not well understood, therefore more *in vitro* experiments should be performed to obtain more insights. Also, finasteride is not a specific inhibitor of FMO3 and it may play profoundly protective roles irrespective of decreasing TMAO production because it only decreases TMAO by only 20% but has much stronger impacts on kidney injury and circulating lipids. This effect may be achieved by regulating intestinal microbiota, and *A. muciniphila* might be a core species worthy of attention.

In this study, we demonstrated that HFD-induced hyperlipidemia exacerbated CKD progression characterized by the exacerbated kidney injury, deterioration of systematic inflammation, gut barrier disruption as well as intestinal microbiota dysfunction probably through elevation of serum TMAO level. The beneficial effects of finasteride on HFD-exacerbated CKD could attribute to TMAO decline *via* down-regulation of Fmo3 expression in liver and gut microbiota alteration. To summarize, we believe that lipid nephrotoxicity caused by HFD can be alleviated by finasteride *via* inhibition of TMAO synthesis. The promising effects of finasteride also suggest the potential of targeting gut microbiota for renal disease treatment.

## Data Availability

The original contributions presented in the study are publicly available. This data can be found here: https://www.ncbi.nlm.nih.gov/, PRJNA795259.
