# Supplementary figures and images for: Finasteride Alleviates High Fat Associated Protein-Overload Nephropathy by Inhibiting Trimethylamine N-Oxide Synthesis and Regulating Gut Microbiota
Source: Front Physiol. 2022 Aug 15;13:900961. doi: 10.3389/fphys.2022.900961 (PMC9420981; doi:10.3389/fphys.2022.900961)

Ctrl

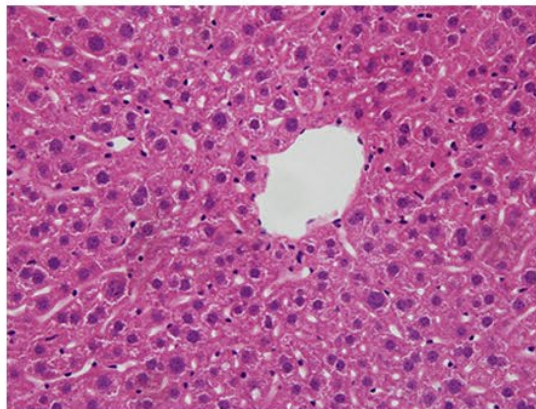

BSA

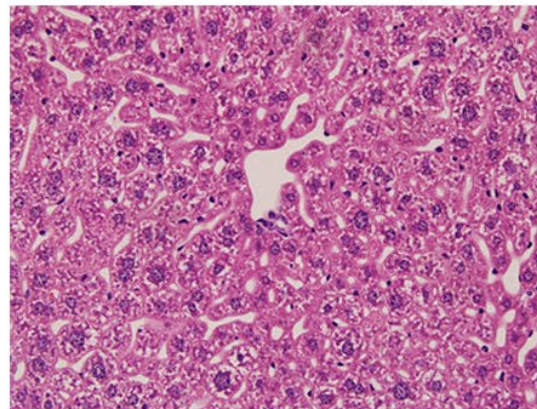

BSA+HFD

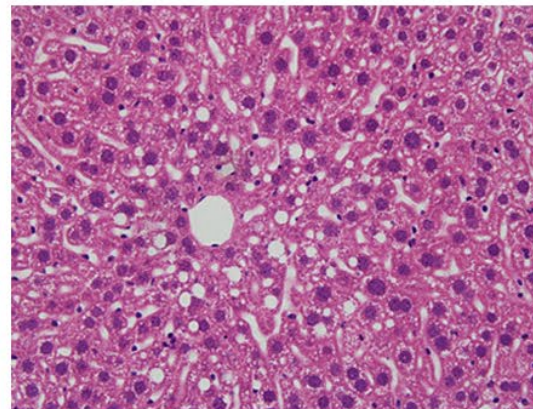

BSA+HFD+Fin

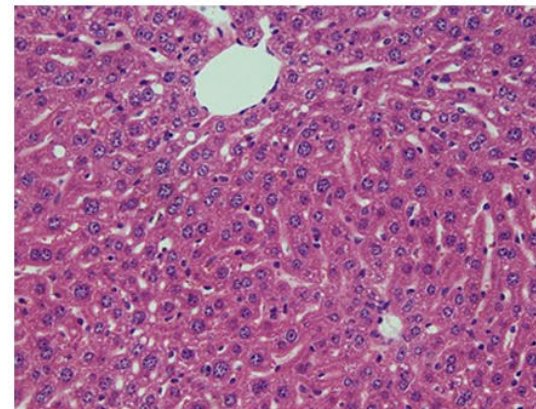

HE-staining

Oil Red O-staining

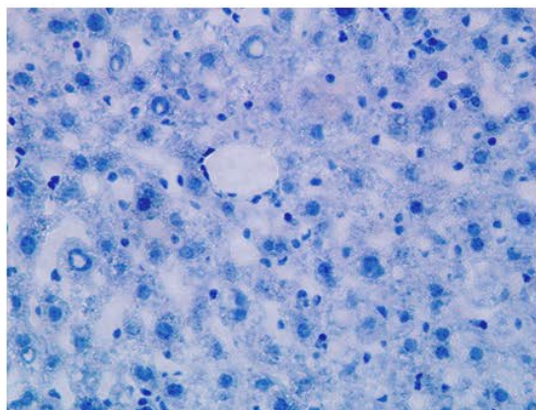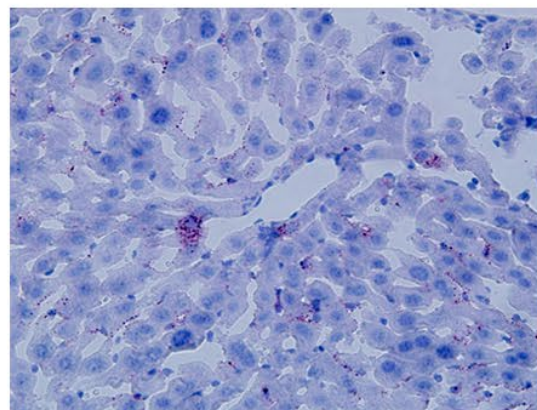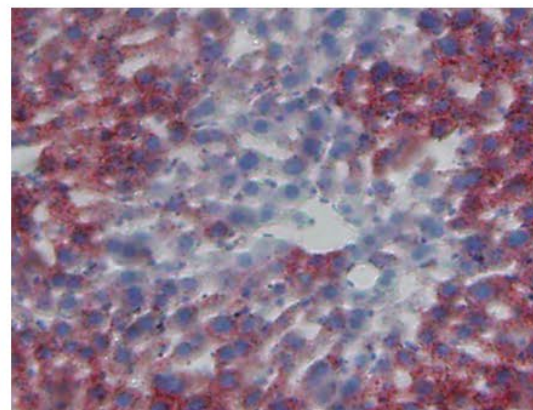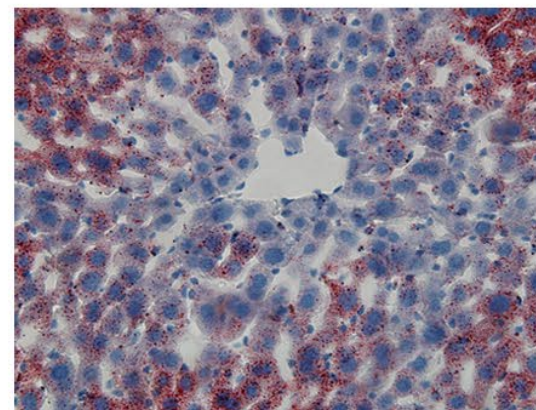

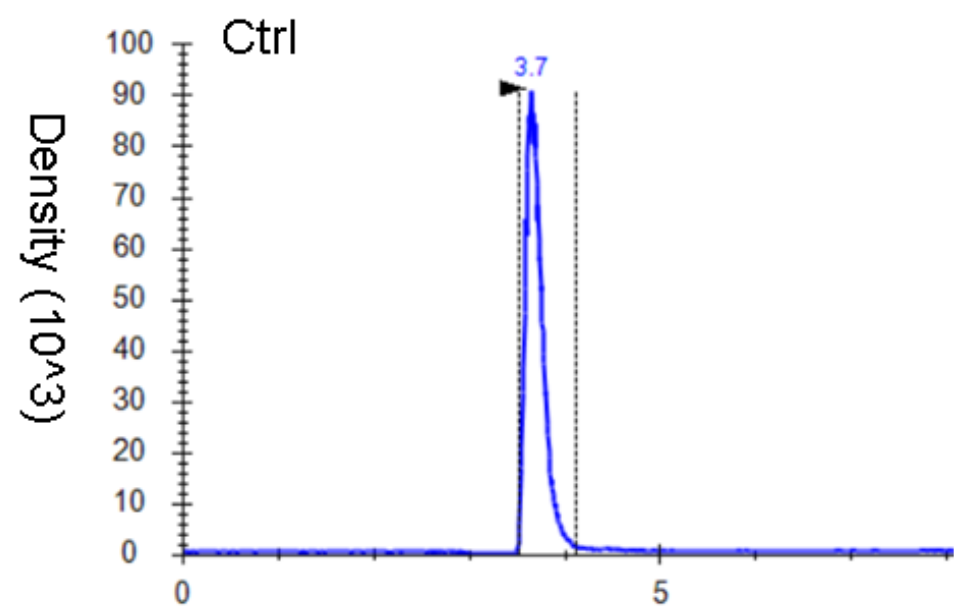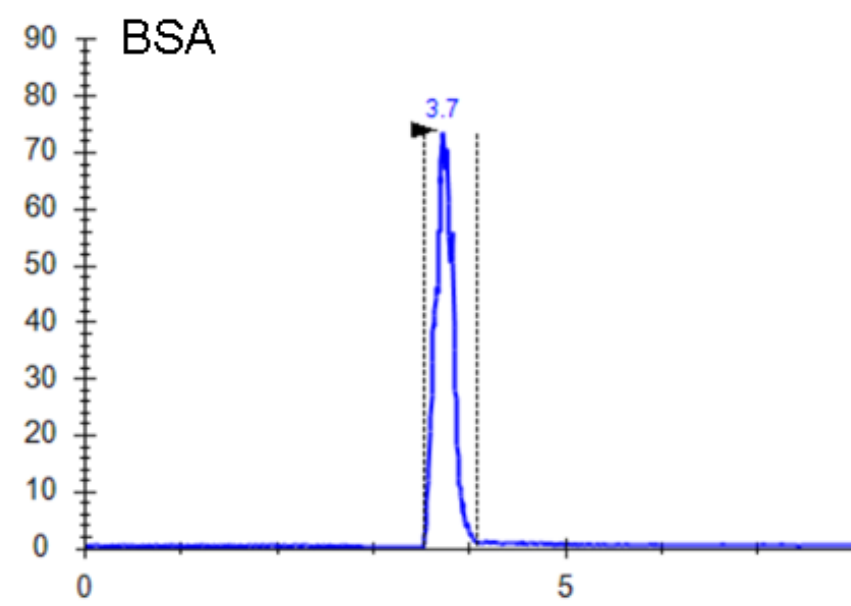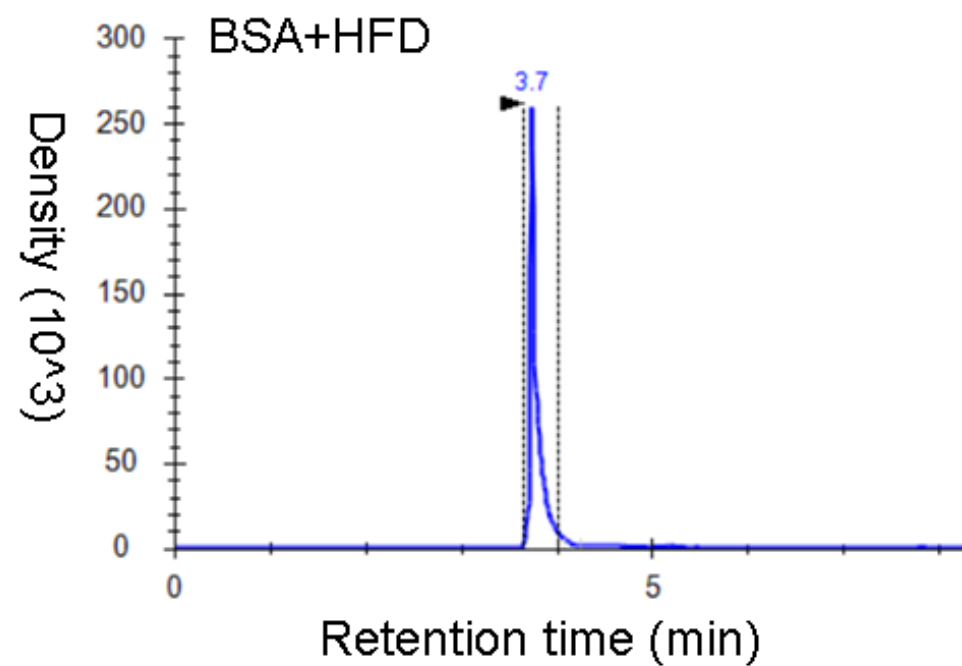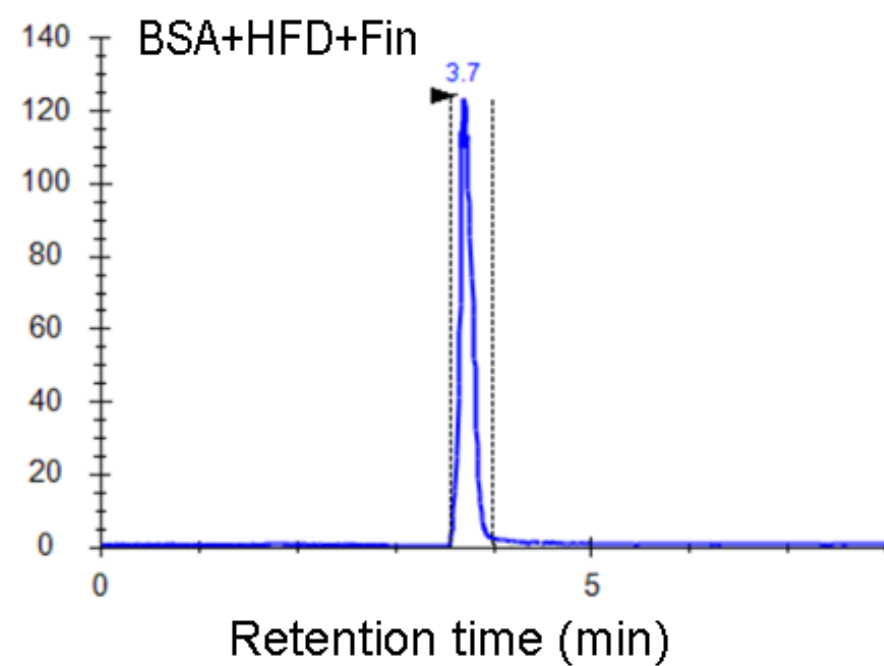

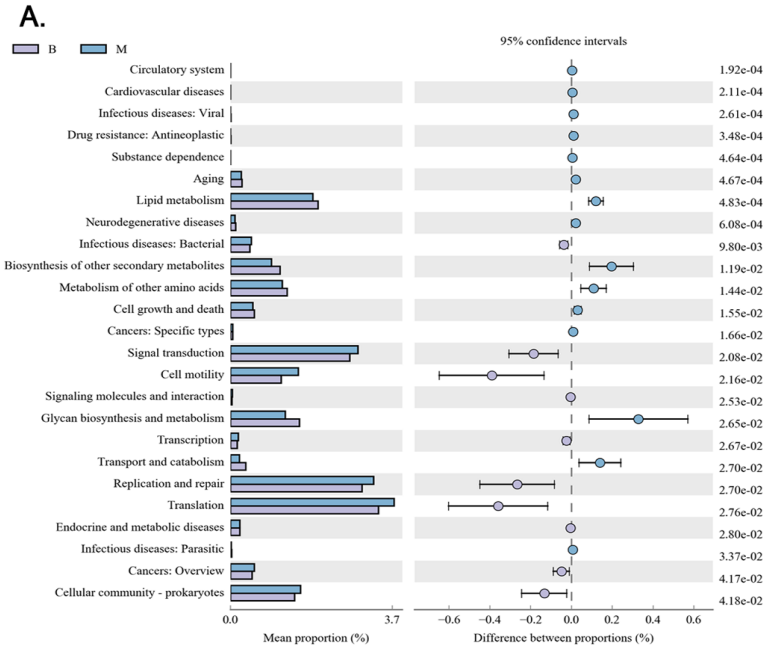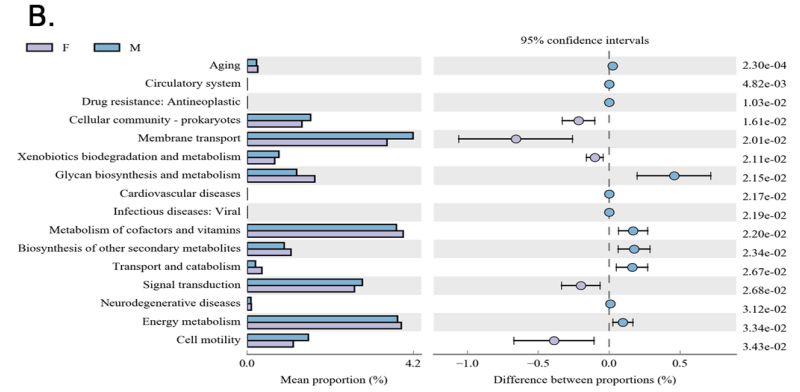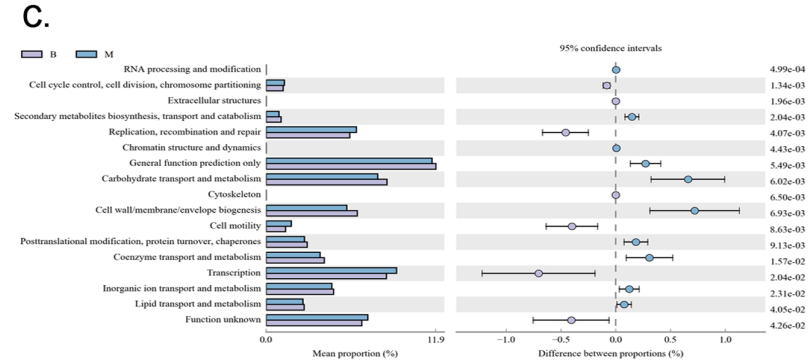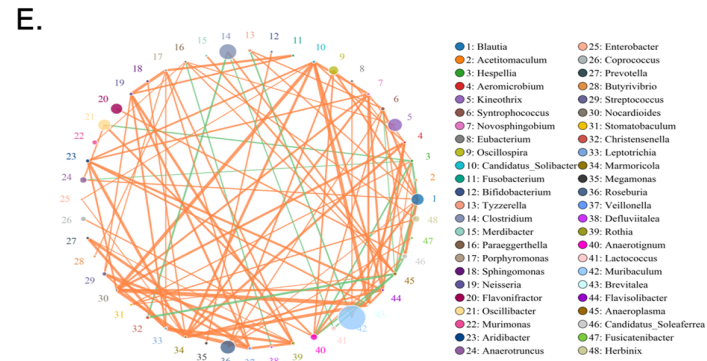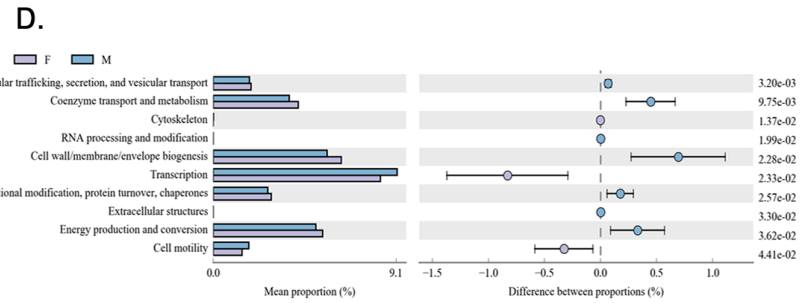

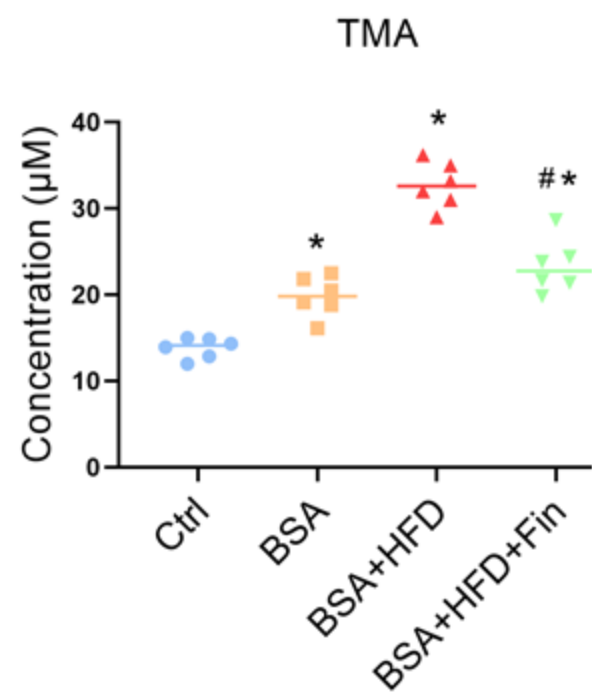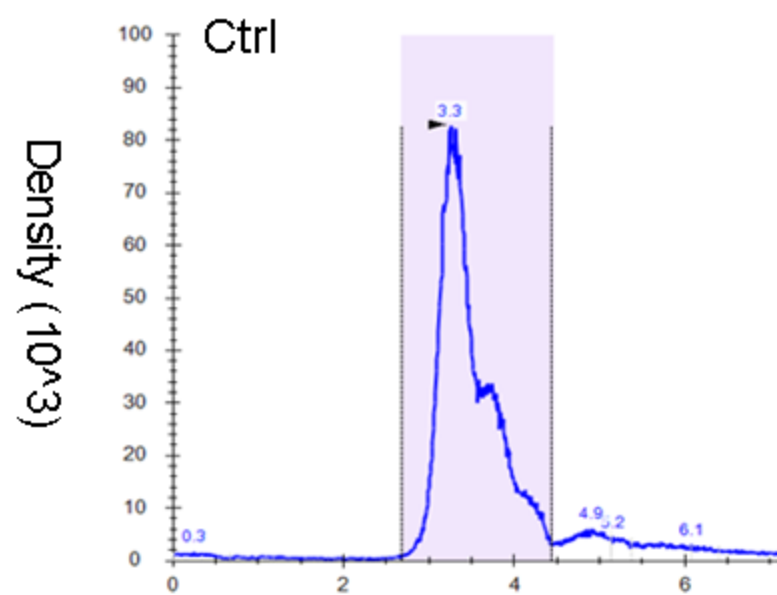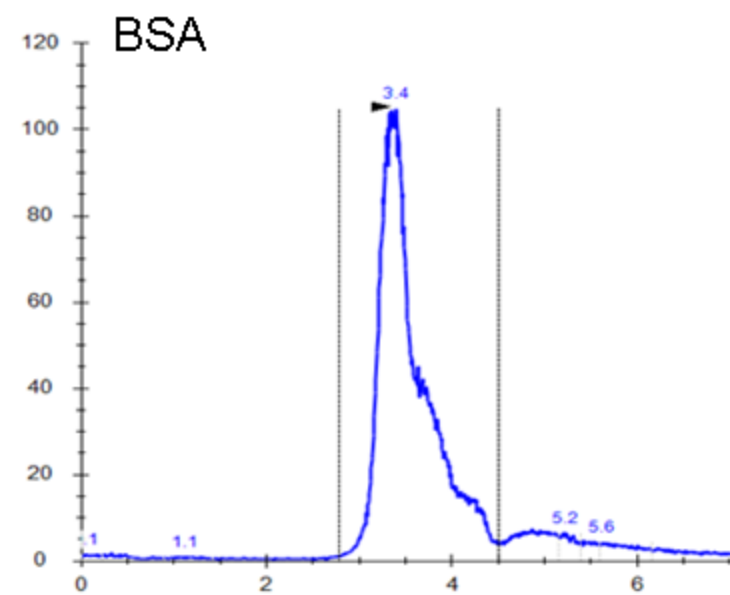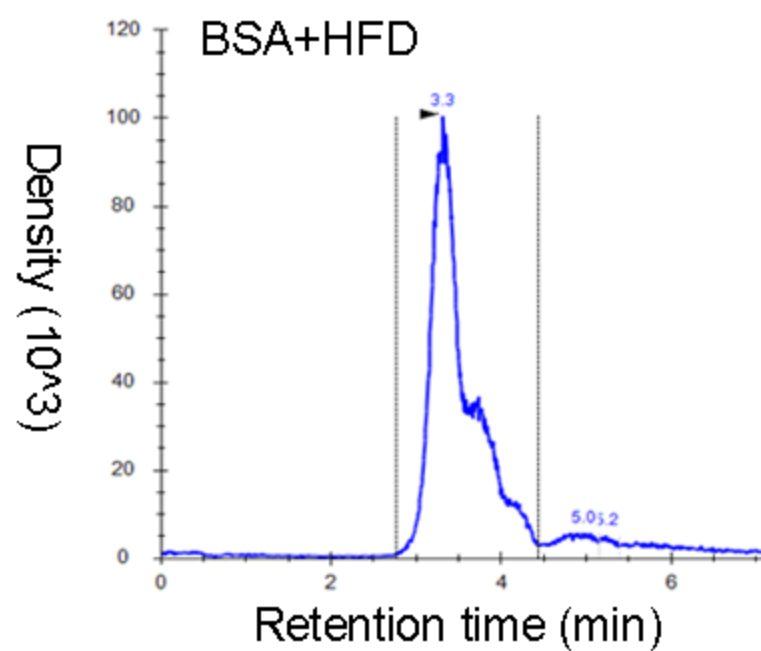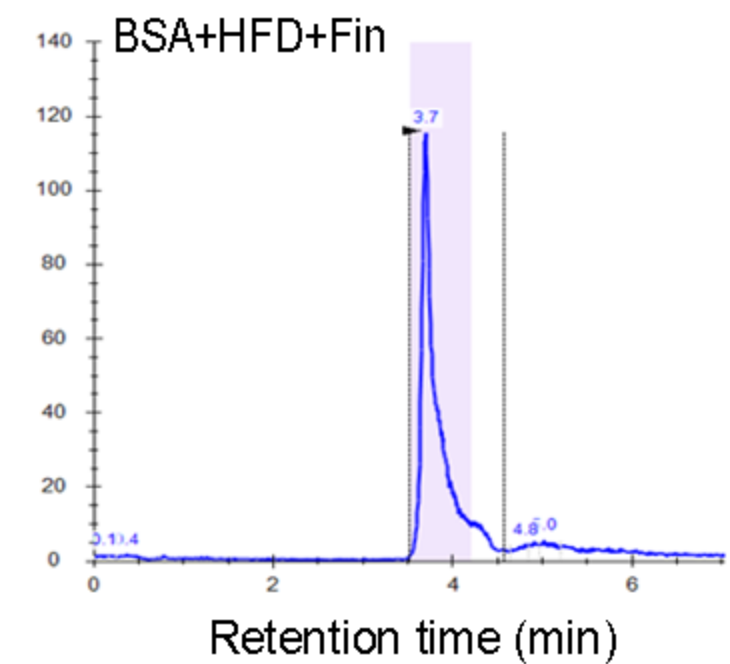

Supplement: Supplementary file 1 [file DataSheet2.PDF]
