# Supplementary material for: Finasteride Alleviates High Fat Associated Protein-Overload Nephropathy by Inhibiting Trimethylamine N-Oxide Synthesis and Regulating Gut Microbiota
Source: Front Physiol. 2022 Aug 15;13:900961. doi: 10.3389/fphys.2022.900961 (PMC9420981; doi:10.3389/fphys.2022.900961)
Supplement: Supplementary file 2 [file DataSheet1.PDF]

Table S1 The results of sample sequencing data processing statistics
Table S2 Alpha diversity index statistics
Table S3 Species statistics of different grades of samples
Table S4 Analysis on the difference of metabolic pathway of KEGG
Table S5 Analysis on the difference of metabolic pathway of COG

Table S1

Sample ID	Raw Reads	Clean Reads	Effective Reads	AvgLen(bp)	GC(%)	Q20(%)	Q30(%)	Effective(%)
B1	79893	79674	77396	416	53.42	99.28	96.68	96.87
B2	80286	80026	77312	416	53.44	99.28	96.69	96.3
B3	80215	79989	77296	414	53.69	99.27	96.68	96.36
B4	79980	79756	77565	415	53.71	99.26	96.66	96.98
B5	79801	79571	76505	415	54.28	99.29	96.75	95.87
B6	80258	80021	77440	415	53.78	99.26	96.68	96.49
B7	79881	79628	76702	416	53.72	99.26	96.65	96.02
B8	79839	79608	76899	413	54.03	99.28	96.7	96.32
C1	80388	80097	77450	416	52.09	98.99	96.01	96.35
C2	55487	55304	51347	412	53.91	99.04	96.18	92.54
C3	79899	79673	75718	412	54.27	99.08	96.27	94.77
C4	79920	79675	76568	414	53.13	99.03	96.1	95.81
C5	79881	79602	74125	416	53.2	98.98	96	92.79
C6	79935	79667	77196	416	53.18	99.02	96.08	96.57
C7	75342	75100	72397	415	53.15	99.02	96.1	96.09
C8	79938	79700	77143	412	53.96	99.04	96.16	96.5
F1	79748	79477	75713	417	53.02	98.98	95.97	94.94
F3	80001	79759	77371	415	52.74	98.99	96.03	96.71
F4	79921	79679	77160	417	54.04	98.96	95.96	96.55
F5	65891	65674	61412	415	52.33	99	96.04	93.2
F6	79986	79752	77609	416	52.46	98.97	95.93	97.03
F7	79893	79651	75452	414	53.03	99	96.06	94.44
F8	80187	79913	74987	413	53.41	98.99	96.05	93.52
F2	56273	56092	53402	414	53.55	99.01	96.07	94.9
M1	80030	79762	76256	414	52.53	99.01	96.1	95.28
M2	80131	79866	76834	412	54.12	99.04	96.16	95.89
M3	79952	79711	76877	417	53.09	98.93	95.84	96.15
M4	80189	79928	77541	409	54.8	99.06	96.24	96.7
M7	80552	80289	77699	415	53.74	99	96.07	96.46
M6	80102	79815	76659	413	54.39	98.98	96	95.7
M5	80192	79886	77168	412	54.65	99	96.07	96.23

Table S2

Sample ID	Feature	ACE	Chao1	Shannon	PD_whole_tree	Coverage
B1	509	527.5563	536.7895	6.3723	28.2251	0.9995
B2	491	505.1213	507.9167	5.9745	27.4375	0.9996
B3	521	541.9863	556.2857	6.282	28.9601	0.9994
B4	514	522.5881	522.8846	6.0373	28.3371	0.9997
B5	517	529.7699	528.8125	6.2696	28.0505	0.9996
B6	515	523.4543	522.7778	6.3813	28.7412	0.9997
B7	526	545.0092	559.0556	6.5485	29.0839	0.9994
B8	511	523.5631	538	6.5529	28.0221	0.9996
C1	433	456.8908	468.037	5.6611	24.3611	0.9994
C2	454	473.476	502.75	6.8331	25.7071	0.9989
C3	452	464.7598	479	6.7291	25.0057	0.9995
C4	456	462.398	466.9286	6.8148	25.6117	0.9997
C5	460	467.3627	470	6.3138	25.4542	0.9996
C6	460	469.1701	473.8	6.3179	25.1176	0.9996
C7	453	467.913	468.12	6.5931	24.6837	0.9996
C8	449	463.0181	467.4545	6.5547	24.6869	0.9996
F1	364	403.6257	445.0526	4.9234	21.9266	0.9992
F2	365	385.7733	391.64	6.078	22.3003	0.9992
F3	373	402.7263	404.9355	5.7197	23.2639	0.9994
F4	360	387.7773	392.25	5.599	22.2094	0.9994
F5	352	381.422	390.0769	5.5186	21.0611	0.9992
F6	332	364.302	359.7742	5.169	20.4128	0.9994
F7	363	387.1161	390.0385	5.7898	22.4359	0.9994
F8	371	399.9241	408.84	5.7572	23.5581	0.9993
M1	361	379.2472	383	5.5488	21.9145	0.9995
M2	366	400.3483	394.75	5.7832	22.0155	0.9993
M3	343	380.8746	373.8857	5.0609	21.0328	0.9993
M4	345	381.6795	404.3684	5.9181	20.331	0.9993
M5	382	410.7614	429.3	6.2557	23.3722	0.9993
M6	367	401.6972	416	5.9307	22.2322	0.9993
M7	344	362.6859	373.5263	5.8006	21.3178	0.9995

Table S3

Sample	Kindom	Phylum	Class	Order	Family	Genus	Species
B1	56528	56528	56528	56528	56528	56528	56528
B2	60775	60775	60775	60775	60775	60775	60775
B3	55951	55951	55951	55951	55951	55951	55951
B4	59117	59117	59117	59117	59117	59117	59117
B5	60356	60356	60356	60356	60356	60356	60356
B6	55892	55892	55892	55892	55892	55892	55892
B7	55336	55336	55336	55336	55336	55336	55336
B8	57244	57244	57244	57244	57244	57244	57244
C1	45997	45997	45997	45997	45997	45997	45997
C2	33537	33537	33537	33537	33537	33537	33537
C3	50960	50960	50960	50960	50960	50960	50960
C4	58469	58469	58469	58469	58469	58469	58469
C5	51452	51452	51452	51452	51452	51452	51452
C6	54399	54399	54399	54399	54399	54399	54399
C7	56170	56170	56170	56170	56170	56170	56170
C8	56898	56898	56898	56898	56898	56898	56898
F1	63543	63543	63543	63543	63543	63543	63543
F2	44979	44979	44979	44979	44979	44979	44979
F3	64774	64774	64774	64774	64774	64774	64774
F4	65698	65698	65698	65698	65698	65698	65698
F5	50778	50778	50778	50778	50778	50778	50778
F6	72499	72499	72499	72499	72499	72499	72499
F7	59497	59497	59497	59497	59497	59497	59497
F8	59877	59877	59877	59877	59877	59877	59877
M1	64664	64664	64664	64664	64664	64664	64664
M2	66159	66159	66159	66159	66159	66159	66159
M3	67290	67290	67290	67290	67290	67290	67290
M4	68049	68049	68049	68049	68049	68049	68049
M5	61526	61526	61526	61526	61526	61526	61526
M6	64467	64467	64467	64467	64467	64467	64467
M7	60027	60027	60027	60027	60027	60027	60027

Table S4

KEGG pathway analysis B versus M										
Class1	Class2	B: mean rel.freq.(%)	B: std.dev.(%)	M: mean rel.freq.(%)	M: std.dev.(%)	p-values	p-values (corrected)	Difference between means	95.0% lower CI	95.0% upper CI
Metabolism	Carbohydrate metabolism	10.2889	0.175395	9.938481	0.346061	0.052791	0.08994	0.350422	-0.00528	0.706127
Metabolism	Lipid metabolism	2.00818	0.01323	1.888425	0.035247	7.34E-05	0.000483	0.119755	0.084164	0.155346
Metabolism	Metabolism of cofactors and vitamins	3.873408	0.064233	3.776395	0.098928	0.066528	0.109296	0.097013	-0.00798	0.202008
Metabolism	Energy metabolism	3.848142	0.065726	3.802149	0.056122	0.196654	0.266062	0.045993	-0.02702	0.119003
Metabolism	Amino acid metabolism	6.603265	0.085383	6.487996	0.288008	0.376355	0.467901	0.11527	-0.17385	0.404392
Metabolism	Nucleotide metabolism	3.648136	0.067016	3.812774	0.192922	0.085542	0.135687	-0.16464	-0.35897	0.029694
Metabolism	Biosynthesis of other secondary metabolites	1.134329	0.059562	0.938071	0.103874	0.002585	0.011893	0.196259	0.088061	0.304457
Metabolism	Metabolism of terpenoids and polyketides	1.082814	0.010769	1.091564	0.025285	0.453521	0.52155	-0.00875	-0.03442	0.016924
Metabo	Xenobioti	0.807	0.034	0.803	0.056	0.87	0.961	0.004	-	0.06

lism	cs biodegradation and metabolism	761	422	562	885	7939	552	199	0.05549	389
Metabolism	Metabolism of other amino acids	1.297113	0.025222	1.189166	0.061301	0.004064	0.014381	0.107947	0.045811	0.170083
Metabolism	Glycan biosynthesis and metabolism	1.580543	0.161172	1.252346	0.223882	0.012691	0.026536	0.328197	0.085623	0.57077
Genetic Information Processing	Translation	3.385323	0.070583	3.744596	0.242316	0.010203	0.027607	-0.35927	-0.60246	-0.11609
Metabolism	Global and overview maps	41.72052	0.183552	41.46409	0.661792	0.389421	0.471405	0.256429	-0.40728	0.920135
Human Diseases	Drug resistance: Antimicrobial	0.958362	0.011355	0.959754	0.029392	0.915886	0.979785	-0.00139	-0.0311	0.028319
Human Diseases	Drug resistance: Antineoplastic	0.009435	0.002268	0.000415	6.53E-05	1.51E-05	0.000348	0.00902	0.006993	0.011047
Environmental Information Processing	Membrane transport	3.970779	0.312989	4.194755	0.361153	0.259079	0.331046	-0.22398	-0.63599	0.188033
Environmental Information Process	Signal transduction	2.727533	0.075741	2.913044	0.112771	0.006321	0.02077	-0.18551	-0.30594	-0.06508

ing										
Cellular Process es	Cellular communit y - prokaryot es	1.471 804	0.076 585	1.605 109	0.101 088	0.02 2715	0.041 795	- 0.133 3	- 0.24 418	- 0.02 243
Cellular Process es	Cell motility	1.163 938	0.149 999	1.554 622	0.244 031	0.00 7027	0.021 55	- 0.390 68	- 0.64 736	- 0.13 401
Genetic Informa tion Process ing	Folding, sorting and degradati on	1.505 916	0.011 652	1.547 686	0.068 771	0.18 9743	0.264 49	- 0.041 77	- 0.11 052	0.02 6985
Genetic Informa tion Process ing	Transcript ion	0.157 629	0.004 881	0.182 088	0.016 955	0.01 1593	0.026 665	- 0.024 46	- 0.04 147	- 0.00 745
Genetic Informa tion Process ing	Replicatio n and repair	3.011 178	0.043 545	3.278 024	0.182 76	0.01 1136	0.026 961	- 0.266 85	- 0.44 985	- 0.08 384
Organis mal System s	Endocrine system	0.557 57	0.014 169	0.545 164	0.021 136	0.24 9277	0.327 621	0.012 407	- 0.01 016	0.03 4971
Environ mental Informa tion Process ing	Signaling molecules and interactio n	0.040 343	0.000 801	0.044 453	0.002 688	0.00 8797	0.025 29	- 0.004 11	- 0.00 681	- 0.00 141
Cellular Process es	Cell growth and death	0.546 782	0.010 141	0.516 086	0.017 481	0.00 4042	0.015 496	0.030 696	0.01 246	0.04 8932
Cellular Process es	Transport and catabolis m	0.350 69	0.063 706	0.211 369	0.095 661	0.01 2307	0.026 958	0.139 321	0.03 7325	0.24 1317
Organis mal System	Aging	0.267 594	0.007 07	0.247 188	0.005 546	6.09 E-05	0.000 467	0.020 406	0.01 2834	0.02 7979

s										
Organismal Systems	Circulatory system	0.002174	0.000517	0.000256	5.94E-05	2.09E-05	0.000192	0.001918	0.001455	0.002381
Organismal Systems	Immune system	0.082548	0.004104	0.083562	0.010642	0.831812	0.933253	-0.00101	-0.01177	0.009742
Organismal Systems	Environmental adaptation	0.183309	0.004144	0.196558	0.015822	0.08825	0.135316	-0.01325	-0.02911	0.002608
Organismal Systems	Nervous system	0.225653	0.00828	0.22112	0.011978	0.452339	0.533528	0.004533	-0.00833	0.017398
Human Diseases	Endocrine and metabolic diseases	0.212509	0.004108	0.217804	0.001089	0.01096	0.028008	-0.0053	-0.00901	-0.00158
Organismal Systems	Excretory system	0.021386	0.002839	0.024652	0.003791	0.11085	0.164487	-0.00327	-0.00741	0.00088
Organismal Systems	Digestive system	0.040137	0.011891	0.022768	0.015428	0.045938	0.081275	0.01737	0.000373	0.034366
Human Diseases	Neurodegenerative diseases	0.124995	0.007994	0.104375	0.004866	0.000106	0.000608	0.02062	0.012726	0.028514
Human Diseases	Substance dependence	0.004945	0.00116	3.96E-05	2.63E-05	1.01E-05	0.000464	0.004905	0.003869	0.005942
Human Diseases	Infectious diseases: Bacterial	0.443933	0.010817	0.481889	0.019157	0.001917	0.009796	-0.03796	-0.05787	-0.01804
Human Diseases	Infectious diseases: Parasitic	0.027965	0.002241	0.021447	0.004906	0.016867	0.033734	0.006518	0.001516	0.01152
Human Diseases	Infectious diseases: Viral	0.009403	0.002367	0.000143	3.77E-05	1.70E-05	0.000261	0.00926	0.007144	0.011375

Human Diseases	Cancers: Overview	0.498625	0.006886	0.548232	0.039633	0.02178	0.041746	-0.04961	-0.08923	-0.00998
Human Diseases	Cancers: Specific types	0.058301	0.004393	0.051196	0.002708	0.00398	0.016643	0.007104	0.002753	0.011456
Human Diseases	Immune diseases	0.042593	0.00174	0.036535	0.009406	0.168203	0.241792	0.006058	-0.00335	0.015464
Human Diseases	Cardiovascular diseases	0.003531	0.0009	4.90E-05	1.32E-05	1.83E-05	0.000211	0.003482	0.002677	0.004287
KEGG pathway analysis M versus F										
Class1	Class2	F: mean rel.freq.(%)	F: std.dev.(%)	M: mean rel.freq.(%)	M: std.dev.(%)	p-values	p-values (corrected)	Difference between means	95.0% lower CI	95.0% upper CI
Metabolism	Carbohydrate metabolism	10.05312	0.198214	9.938481	0.346061	0.491214	0.57938	0.114636	-0.24578	0.475053
Metabolism	Lipid metabolism	1.919101	0.027641	1.888425	0.035247	0.11171	0.22342	0.030677	-0.00833	0.069688
Metabolism	Metabolism of cofactors and vitamins	3.944916	0.05416	3.776395	0.098928	0.004784	0.022005	0.168521	0.066043	0.270999
Metabolism	Energy metabolism	3.898481	0.062172	3.802149	0.056122	0.011625	0.033423	0.096333	0.02541	0.167255
Metabolism	Amino acid metabolism	6.626336	0.093698	6.487996	0.288008	0.296611	0.379003	0.13834	-0.1513	0.427978
Metabolism	Nucleotide metabolism	3.696483	0.094857	3.812774	0.192922	0.214034	0.317599	-0.11629	-0.31407	0.08149
Metabolism	Biosynthesis of	1.113861	0.074132	0.938071	0.103874	0.005605	0.023439	0.17579	0.063462	0.288118

	other secondary metabolites									
Metabolism	Metabolism of terpenoids and polyketides	1.064706	0.011384	1.091564	0.025285	0.042854	0.098565	-0.02686	-0.05262	-0.0011
Metabolism	Xenobiotics biodegradation and metabolism	0.701922	0.03406	0.803562	0.056885	0.003667	0.021087	-0.10164	-0.16124	-0.04204
Metabolism	Metabolism of other amino acids	1.222525	0.024944	1.189166	0.061301	0.248897	0.357789	0.033359	-0.02874	0.095463
Metabolism	Glycan biosynthesis and metabolism	1.709173	0.20358	1.252346	0.223882	0.002339	0.021523	0.456827	0.19709	0.716564
Genetic Information Processing	Translation	3.58609	0.129092	3.744596	0.242316	0.185203	0.293771	-0.15851	-0.40878	0.091767
Metabolism	Global and overview maps	42.06301	0.26861	41.46409	0.661792	0.073098	0.160119	0.598912	-0.07146	1.269286
Human Diseases	Drug resistance: Antimicrobial	0.974335	0.008876	0.959754	0.029392	0.280499	0.379499	0.014581	-0.01493	0.044096
Human Diseases	Drug resistance: Antineoplastic	0.000749	0.000163	0.000415	6.53E-05	0.00067	0.010274	0.000334	0.00183	0.000485

Environmental Information Processing	Membrane transport	3.536166	0.285659	4.194755	0.361153	0.003939	0.020132	-0.65859	-1.05926	-0.25792
Environmental Information Processing	Signal transduction	2.712489	0.111492	2.913044	0.112771	0.007003	0.026845	-0.20055	-0.33577	-0.06534
Cellular Processes	Cellular community - prokaryotes	1.389109	0.083058	1.605109	0.101088	0.001402	0.016119	-0.216	-0.32939	-0.10261
Cellular Processes	Cell motility	1.165893	0.22227	1.554622	0.244031	0.011192	0.034324	-0.38873	-0.67201	-0.10545
Genetic Information Processing	Folding, sorting and degradation	1.527924	0.029997	1.547686	0.068771	0.532411	0.612272	-0.01976	-0.08968	0.050159
Genetic Information Processing	Transcription	0.168846	0.007661	0.182088	0.016955	0.115261	0.203923	-0.01324	-0.03052	0.004034
Genetic Information Processing	Replication and repair	3.144774	0.085697	3.278024	0.18276	0.13898	0.236781	-0.13325	-0.31993	0.053431
Organismal Systems	Endocrine system	0.563967	0.013664	0.545164	0.021136	0.091113	0.19051	0.018804	-0.00361	0.041219
Environmental Information Process	Signaling molecules and interaction	0.043077	0.001396	0.044453	0.002688	0.288668	0.379392	-0.00138	-0.00415	0.001393

ing										
Cellular Processes	Cell growth and death	0.527777	0.005542	0.516086	0.017481	0.159762	0.262466	0.011691	-0.00588	0.029261
Cellular Processes	Transport and catabolism	0.372619	0.083942	0.211369	0.095661	0.007538	0.026673	0.16125	0.051658	0.270841
Organismal Systems	Aging	0.272082	0.006539	0.247188	0.005546	5.00E-06	0.00023	0.024894	0.017653	0.032135
Organismal Systems	Circulatory system	0.000485	9.59E-05	0.000256	5.94E-05	0.00021	0.004822	0.000229	0.000134	0.000324
Organismal Systems	Immune system	0.092125	0.005815	0.083562	0.010642	0.112645	0.215903	0.008563	-0.00246	0.019584
Organismal Systems	Environmental adaptation	0.188661	0.010936	0.196558	0.015822	0.326405	0.4058	-0.0079	-0.02489	0.009096
Organismal Systems	Nervous system	0.23068	0.00651	0.22112	0.011978	0.114975	0.211555	0.00956	-0.00284	0.021958
Human Diseases	Endocrine and metabolic diseases	0.217466	0.001254	0.217804	0.001089	0.6113	0.685849	-0.00034	-0.00174	0.001065
Organismal Systems	Excretory system	0.02921	0.00331	0.024652	0.003791	0.040875	0.09896	0.004558	0.000223	0.008894
Organismal Systems	Digestive system	0.040453	0.010924	0.022768	0.015428	0.039439	0.100788	0.017685	0.00103	0.03434
Human Diseases	Neurodegenerative diseases	0.111655	0.002158	0.104375	0.004866	0.009499	0.031212	0.00728	0.002328	0.012232
Human	Substance	6.85E	4.85E	3.96E	2.63E	0.19	0.306	2.90	####	7.56

Diseases	dependence	-05	-05	-05	-05	9692	195	E-05	##	E-05
Human Diseases	Infectious diseases: Bacterial	0.456628	0.013582	0.481889	0.019157	0.021269	0.057552	-0.02526	-0.04595	-0.00458
Human Diseases	Infectious diseases: Parasitic	0.020373	0.002411	0.021447	0.004906	0.637882	0.698633	-0.00107	-0.0061	0.003955
Human Diseases	Infectious diseases: Viral	0.000392	0.000154	0.000143	3.77E-05	0.003331	0.021887	0.000249	0.00011	0.000388
Human Diseases	Cancers: Overview	0.527158	0.012052	0.548232	0.039633	0.250454	0.349118	-0.02107	-0.06088	0.018729
Human Diseases	Cancers: Specific types	0.051497	0.002099	0.051196	0.002708	0.828755	0.886575	0.000301	-0.00269	0.003289
Human Diseases	Immune diseases	0.033491	0.004049	0.036535	0.009406	0.482917	0.584584	-0.00304	-0.0126	0.006513
Human Diseases	Cardiovascular diseases	0.000135	5.16E-05	4.90E-05	1.32E-05	0.002833	0.021719	8.55E-05	3.90E-05	0.000132

Table S5

COG analysis B versus M										
Class1	Class2	B: mean rel.freq.(%)	B: std.dev.(%)	M: mean rel.freq.(%)	M: std.dev.(%)	p-values	p-values (corrected)	Difference between means	95.0% lower CI	95.0% upper CI
INFORMATION STORAGE AND PROCESSING	RNA processing and modification	0.001022	0.000219	0.00023	4.05E-05	2.00E-05	0.000499	0.000792	0.000595	0.000989
INFORMATION STORAGE AND PROCESSING	Chromatin structure and dynamics	0.007321	0.001674	0.002477	0.00215	0.000887	0.004433	0.004843	0.002468	0.007218
METABOLISM	Energy production and conversion	5.321154	0.135481	5.108243	0.218875	0.066456	0.087443	0.212911	-0.01755	0.443375
CELLULAR PROCESSES AND SIGNALING	Cell cycle control, cell division, chromosome partitioning	1.202167	0.021196	1.285917	0.028697	0.000107	0.001344	-0.08375	-0.11503	-0.05247
METABOLISM	Amino acid transport and metabolism	8.900461	0.075351	8.972436	0.218047	0.46567	0.52917	-0.07197	-0.29158	0.147633
METABOLISM	Nucleotide transport and metabolism	3.363697	0.047235	3.400291	0.14217	0.565414	0.61458	-0.03659	-0.17964	0.10645
METABOLISM	Carbohydrate transport and metabolism	8.492417	0.170102	7.833842	0.325153	0.001687	0.006025	0.658575	0.323361	0.993788
METABO	Coenzyme	4.093	0.162	3.786	0.184	0.00	0.01	0.30	0.09	0.51

LISM	transport and metabolism	737	77	697	264	8168	5708	704	5458	8622
METABOLISM	Lipid transport and metabolism	2.676871	0.068127	2.601342	0.036601	0.02755	0.040514	0.075528	0.010036	0.14102
INFORMATION STORAGE AND PROCESSING	Translation, ribosomal structure and biogenesis	7.911302	0.077819	8.270512	0.327472	0.036152	0.050211	-0.35921	-0.68711	-0.03131
INFORMATION STORAGE AND PROCESSING	Transcription	8.442084	0.384656	9.147122	0.454476	0.011428	0.020407	-0.70504	-1.21952	-0.19055
INFORMATION STORAGE AND PROCESSING	Replication, recombination and repair	5.87753	0.092984	6.336636	0.205872	0.000976	0.004066	-0.45911	-0.66886	-0.24935
CELLULAR PROCESSES AND SIGNALING	Cell wall/membrane/envelope biogenesis	6.390938	0.294414	5.672205	0.366722	0.002494	0.006927	0.718732	0.310172	1.127292
CELLULAR PROCESSES AND SIGNALING	Cell motility	1.367821	0.13194	1.7674	0.22512	0.003799	0.008633	-0.39958	-0.63474	-0.16441
CELLULAR PROCESSES AND SIGNALING	Posttranslational modification, protein turnover, chaperones	2.887197	0.046392	2.703559	0.10594	0.00438	0.009126	0.183638	0.075902	0.291374
METABO	Inorganic	4.741	0.032	4.618	0.089	0.01	0.02	0.12	0.03	0.21

LISM	ion transport and metabolism	894	447	168	398	3838	3063	3725	3557	3894
METABOLISM	Secondary metabolites biosynthesis, transport and catabolism	1.055811	0.043727	0.909149	0.057391	0.000326	0.002041	0.146662	0.083625	0.2097
POORLY CHARACTERIZED	General function prediction only	11.90605	0.069895	11.6346	0.134841	0.001756	0.005489	0.271458	0.132569	0.410346
POORLY CHARACTERIZED	Function unknown	6.726577	0.137902	7.133385	0.342963	0.027232	0.042551	-0.40681	-0.75407	-0.05954
CELLULAR PROCESSES AND SIGNALING	Signal transduction mechanisms	4.384017	0.140327	4.55182	0.21784	0.136345	0.170431	-0.1678	-0.39868	0.063079
CELLULAR PROCESSES AND SIGNALING	Intracellular trafficking, secretion, and vesicular transport	1.783134	0.033307	1.805884	0.02077	0.159895	0.190351	-0.02275	-0.05584	0.010342
CELLULAR PROCESSES AND SIGNALING	Defense mechanisms	2.464933	0.042194	2.454239	0.125778	0.847882	0.883211	0.010694	-0.11589	0.137275
CELLULAR PROCESSES AND SIGNALING	Extracellular structures	6.26E-06	2.01E-06	8.64E-05	2.54E-05	0.000236	0.001964	######	-0.00011	######
COG analysis M versus F										
Class1	Class2	F:	F:	M:	M:	p-	p-	Diffe	95.0	95.0

		mean rel.freq.(%)	std.dev.(%)	mean rel.freq.(%)	std.dev.(%)	values	values (corrected)	rence between means	% lower CI	% upper CI
INFORMATION STORAGE AND PROCESSING	RNA processing and modification	0.000342	7.06E-05	0.00023	4.05E-05	0.00398	0.0199	0.000113	4.40E-05	0.000182
INFORMATION STORAGE AND PROCESSING	Chromatin structure and dynamics	0.002969	0.001541	0.002477	0.00215	0.650385	0.70694	0.000491	-0.00184	0.002818
METABOLISM	Energy production and conversion	5.438924	0.168242	5.108243	0.218875	0.01158	0.036187	0.330681	0.089722	0.571641
CELLULAR PROCESSES AND SIGNALING	Cell cycle control, cell division, chromosome partitioning	1.248439	0.027248	1.285917	0.028697	0.032651	0.058305	-0.03748	-0.07132	-0.00363
METABOLISM	Amino acid transport and metabolism	8.925593	0.074291	8.972436	0.218047	0.630774	0.750921	-0.04684	-0.26637	0.172682
METABOLISM	Nucleotide transport and metabolism	3.413978	0.061444	3.400291	0.14217	0.83225	0.866928	0.013687	-0.13079	0.158159
METABOLISM	Carbohydrate transport and metabolism	7.761971	0.194062	7.833842	0.325153	0.646311	0.734444	-0.07187	-0.41239	0.268646
METABOLISM	Coenzyme transport and metabolism	4.232789	0.180516	3.786697	0.184264	0.00078	0.009754	0.446092	0.226006	0.666177

METABOLISM	Lipid transport and metabolism	2.663434	0.051773	2.601342	0.036601	0.026058	0.050111	0.062092	0.008711	0.115472
INFORMATION STORAGE AND PROCESSING	Translation, ribosomal structure and biogenesis	8.345789	0.150902	8.270512	0.327472	0.618282	0.772853	0.075277	-0.25882	0.40937
INFORMATION STORAGE AND PROCESSING	Transcription	8.316867	0.438316	9.147122	0.454476	0.005587	0.023279	-0.83025	-1.3696	-0.2909
INFORMATION STORAGE AND PROCESSING	Replication, recombination and repair	6.172632	0.079663	6.336636	0.205872	0.105823	0.155622	-0.164	-0.37212	0.044115
CELLULAR PROCESSES AND SIGNALING	Cell wall/membrane/envelope biogenesis	6.364862	0.319668	5.672205	0.366722	0.003644	0.022778	0.692656	0.273476	1.111836
CELLULAR PROCESSES AND SIGNALING	Cell motility	1.440338	0.200731	1.7674	0.22512	0.017631	0.044078	-0.32706	-0.5864	-0.06772
CELLULAR PROCESSES AND SIGNALING	Posttranslational modification, protein turnover, chaperones	2.878944	0.084234	2.703559	0.10594	0.007198	0.025706	0.175385	0.057682	0.293089
METABOLISM	Inorganic ion transport and	4.71572	0.073337	4.618168	0.089398	0.055444	0.086631	0.097552	-0.00268	0.197781

	metabolism									
METABOLISM	Secondary metabolites biosynthesis, transport and catabolism	0.945036	0.035534	0.909149	0.057391	0.214332	0.297684	0.035887	-0.02455	0.096319
POORLY CHARACTERIZED	General function prediction only	11.79041	0.098946	11.6346	0.134841	0.039411	0.065685	0.155809	0.009064	0.302553
POORLY CHARACTERIZED	Function unknown	6.703488	0.193237	7.133385	0.342963	0.023225	0.052785	-0.4299	-0.78636	-0.07343
CELLULAR PROCESSES AND SIGNALING	Signal transduction mechanisms	4.25495	0.198877	4.55182	0.21784	0.025128	0.052349	-0.29687	-0.54997	-0.04377
CELLULAR PROCESSES AND SIGNALING	Intracellular trafficking, secretion, and vesicular transport	1.872852	0.024207	1.805884	0.02077	0.000128	0.003197	0.066967	0.040018	0.093916
CELLULAR PROCESSES AND SIGNALING	Defense mechanisms	2.507829	0.054624	2.454239	0.125778	0.361528	0.475695	0.05359	-0.07426	0.18144
CELLULAR PROCESSES AND SIGNALING	Extracellular structures	0.000146	4.51E-05	8.64E-05	2.54E-05	0.011873	0.032979	5.97E-05	1.60E-05	0.000103
